# Curcumin and metabolic syndrome: Mechanistic and clinical evidence for modulating diabetes and cardiovascular risk factors

**DOI:** 10.1016/j.crphar.2026.100262

**Published:** 2026-10-13

**Authors:** Mostafa Vaghari-Tabari, Abdolkarim Mahrooz, Asal Golchin, Mehran Molavand, Vahid Rahnama, Bahman Yousefi

**Affiliations:** a Department of Paramedicine, Amol School of Paramedical Sciences, Mazandaran University of Medical Sciences, Sari, Iran; b Molecular and Cell Biology Research Center, Faculty of Medicine, Mazandaran University of Medical Sciences, Sari, Iran; c Department of Clinical Biochemistry and Laboratory Medicine, Faculty of Medicine, Tabriz University of Medical Science, Tabriz, Iran; d Student Research Committee, Tabriz University of Medical Sciences, Tabriz, Iran

**Keywords:** Curcumin, Metabolic syndrome, Inflammation, Insulin resistance, Bioavailability, Gut microbiota

## Abstract

Metabolic Syndrome (MetS) is characterized by central obesity, hyperglycemia, dyslipidemia, and elevated blood pressure. Obesity contributes to low-grade inflammation, oxidative stress, metabolic endotoxemia, decreased adiponectin, and increased leptin, which play major roles in insulin resistance. Inflammation and insulin resistance enhance fatty acid release from adipose tissue, increasing hepatic uptake, VLDL production, and hypertriglyceridemia. Elevated VLDL further promotes triglyceride–HDL exchange via CETP, lowering HDL levels. Oxidative stress, increased angiotensin II production in adipose tissue, and upregulated receptor expression are also key factors in hypertension associated with MetS. Curcumin, a natural polyphenolic compound, has been shown to modulate many of these processes. Experimental evidence suggests that curcumin can reduce inflammation and oxidative stress, regulate adipokines, improve lipid metabolism, and support vascular health. Several clinical trials have also reported beneficial effects of curcumin supplementation on the components of MetS. In this review, we first summarize the main mechanisms underlying MetS and then discuss in detail the molecular and physiological effects of curcumin on these pathways. Finally, we provide an overview of clinical findings on curcumin and its potential as a supportive strategy in improving MetS.

## Highlights

• Curcumin effectively improves key components of metabolic syndrome including obesity, insulin resistance, dyslipidemia, and hypertension.
• Curcumin reduces inflammation, oxidative stress, and metabolic endotoxemia while modulating adipokines and gut microbiota.
• Advanced formulations (nano-micelle, phytosomal) significantly enhance curcumin bioavailability and clinical efficacy.
• Multi-omics and network pharmacology reveal curcumin’s multi-target actions on AMPK, PPAR, NF-κB, and NRF2 pathways.
• Curcumin shows strong potential as a safe adjunctive nutraceutical for prevention and management of MetS and related cardiometabolic diseases.

## Graphical abstract

**Figure:**
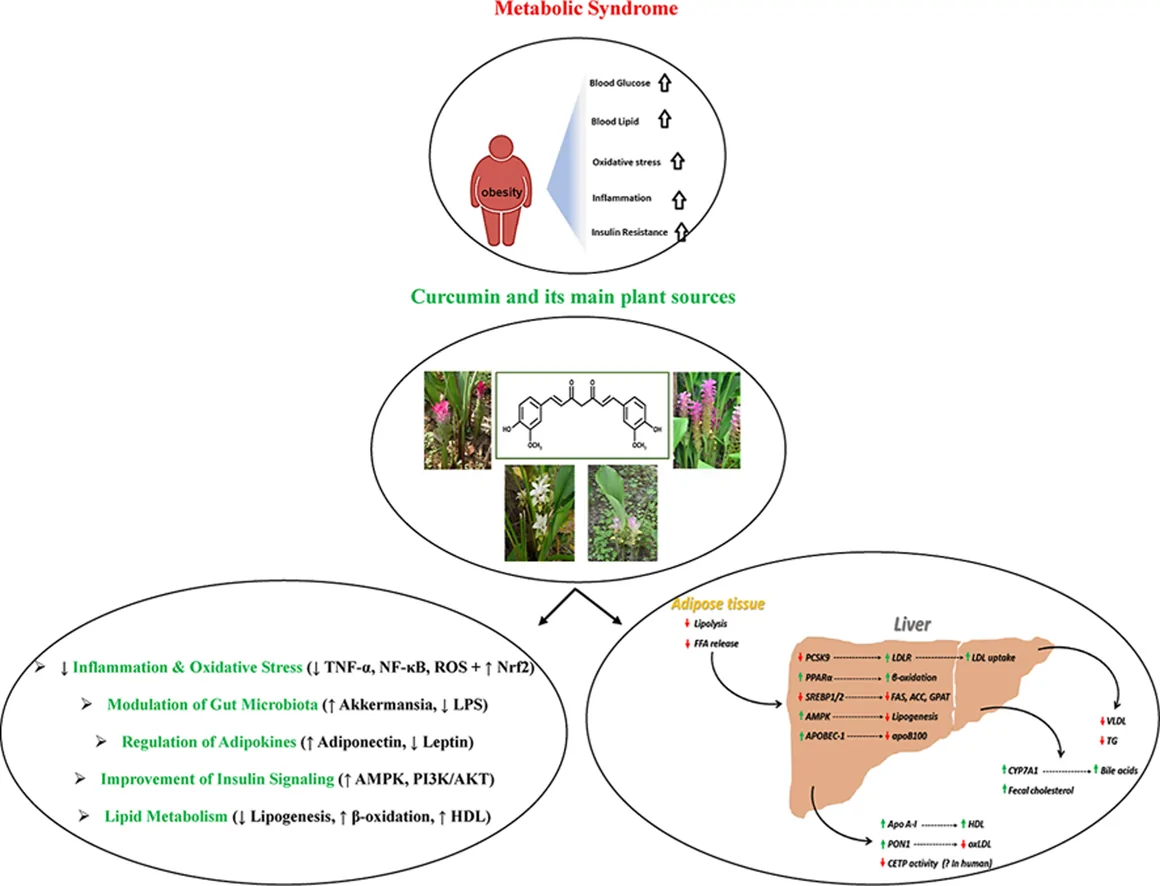

## 1 Introduction

MetS refers to a group of metabolic disorders, including increased fasting plasma glucose (≥100 mg/dL), central obesity, increased blood pressure (systolic ≥130 mmHg or diastolic ≥85 mmHg), increased triglycerides (≥150 mg/dL), and decreased HDL cholesterol (<50 mg/dL in females and <40 mg/dL in males) and is associated with an increased risk of some important diseases, including cardiovascular diseases, type 2 diabetes (T2D), and dementia. The presence of three of the previously mentioned metabolic disorders is a diagnostic criterion for MetS (Nilsson et al., 2019). The worldwide prevalence of MetS ranges from 12.5% to 31.4% (Noubiap et al., 2022). Patients with T2D experience a significantly elevated incidence of MetS. A recent study revealed that the prevalence of MetS in individuals with type 2 diabetes was 78.4% (Essafi et al., 2022). Given that MetS significantly increases the risk of cardiovascular diseases and sudden cardiac death, these statistics are indeed concerning. (Tirandi et al., 2022). Individuals with MetS are at a fivefold increased risk of developing T2D. At present, MetS is managed through a combination of lifestyle modifications and pharmacological treatments. Lifestyle modifications include the following: increasing physical activity, reducing intake of simple carbohydrates and cholesterol, avoiding alcohol and tobacco, managing stress, and ensuring sufficient sleep (Aguilar-Salinas and Viveros-Ruiz, 2019; Regufe et al., 2020; Wang et al., 2020b).

Common drug treatments include metformin to increase insulin sensitivity, statins to improve dyslipidemia, and ACE inhibitors to reduce blood pressure (Aguilar-Salinas and Viveros-Ruiz, 2019). It has become evident that certain natural compounds, particularly dietary polyphenols, can be highly effective in preventing and alleviating MetS. An inverse relationship between the incidence of MetS and dietary polyphenol intake is frequently observed in epidemiological studies. Furthermore, numerous studies have documented the beneficial effects of polyphenols on a variety of parameters that are associated with MetS (Chiva-Blanch and Badimon, 2017). Curcumin, one of the most widely recognized polyphenolic compounds, appears to have beneficial effects on components of MetS (Marina et al., 2025). The primary active ingredient in turmeric (*Curcuma longa*) is curcumin. Curcumin is derived from four primary plant sources: *Curcuma wenyujin*, *Curcuma longa*, *Curcuma phaeocaulis*, and *Curcuma kwangsiensis* (Fig. 1) (Zeng et al., 2023). The U.S. Food and Drug Administration (FDA) has said that curcumin is Generally Recognized as Safe (GRAS) for use in foods and supplements. Curcumin has generally been well tolerated in several short-term human studies, including studies using relatively high oral doses. However, these observations should not be generalized to long-term use or to all formulations and patient populations, because safety may vary according to dose, duration, formulation, systemic exposure, and concomitant treatments (Gupta et al., 2013a; Hewlings and Kalman, 2017; Sharifi-Rad et al., 2020). This polyphenol compound is a compelling object of research for the prevention and treatment of MetS due to its antioxidant, anti-inflammatory, cholesterol-lowering, and anti-diabetic properties (Kocaadam and Şanlier, 2017).

**Fig. 1:**
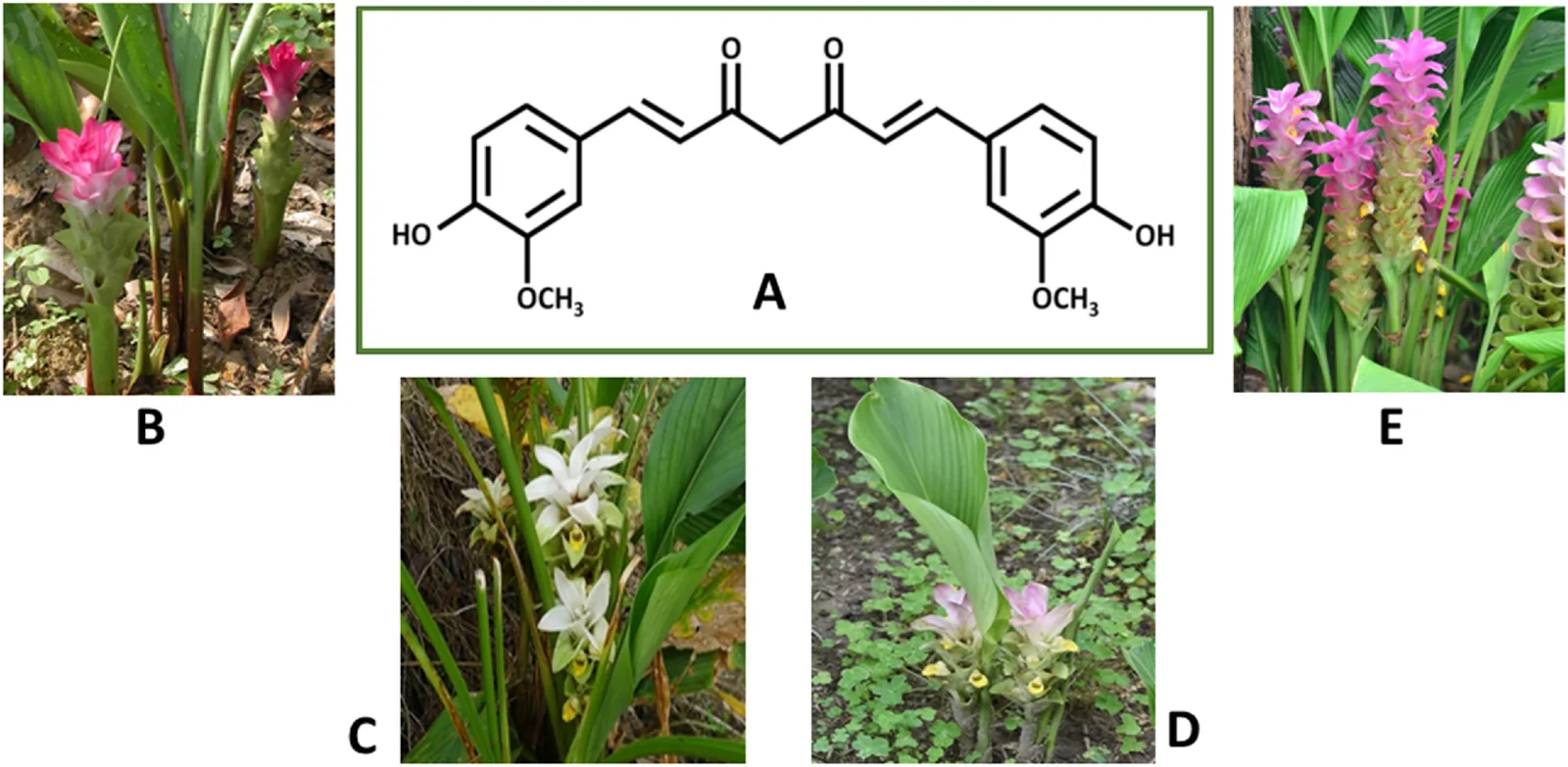
(A) Chemical structure of curcumin and its main plant sources: ((B) *Curcuma kwangsiensis*, (C) *Curcuma longa*, (D) *Curcuma wenyujin*, (E) *Curcuma phaeocaulis*. The pictures are from the website: http://www.iplant.cn/frps, accessed on 5 September 2025.

The distinctive aim of the present structured narrative review is therefore to integrate the pathophysiological architecture of MetS with mechanistic, preclinical, clinical, formulation-specific, and emerging systems-biology evidence on curcumin. Rather than treating AMPK, PPARs, NF-κB, NRF2, PI3K/AKT, eNOS, lipid-regulatory pathways, and the gut microbiota as isolated targets, we examine how their modulation may converge on the interconnected processes of adipose-tissue dysfunction, insulin resistance, dysglycemia, dyslipidemia, chronic inflammation, oxidative stress, and vascular dysfunction. Particular emphasis is placed on distinguishing mechanistic plausibility from demonstrated human effects and on identifying points at which promising preclinical findings have not yet translated into consistent clinical benefit.

## 2 Review methodology and literature search strategy

A structured literature search was performed using PubMed, Scopus, Web of Science from database inception to June 2026. The search strategy combined terms related to curcumin with terms related to MetS and its principal pathophysiological and clinical components. Core search terms included “1,6-Heptadiene-3,5-dione, 1,7-bis(4-hydroxy-3-methoxyphenyl)-, (E,E)-”Turmeric Yellow”, “Curcumin Phytosome”, “Diferuloylmethane”, “metabolic syndrome”, “insulin resistance”, “obesity”, “hyperglycemia”, “type 2 diabetes”, “dyslipidemia”, “hypertriglyceridemia”, “hypertension”, “cardiovascular risk”, “inflammation”, “oxidative stress”, “adipokines”, and “gut microbiota”. Reference lists of relevant original studies, systematic reviews, and meta-analyses were also manually screened to identify additional potentially eligible publications.

Studies were considered eligible if they investigated curcumin, curcuminoids, or a clearly defined curcumin-containing formulation in relation to MetS or outcomes directly relevant to its pathophysiology or clinical components. Eligible evidence included mechanistic in vitro studies, animal studies, human intervention studies, randomized controlled trials, and relevant systematic reviews/meta-analyses. Studies were excluded if they did not investigate curcumin or a clearly characterized curcumin-containing intervention, did not report outcomes relevant to MetS or cardiometabolic risk, were conference abstracts without sufficient methodological information, were editorials or commentaries, or represented duplicate publications without providing additional relevant outcomes.

## 3 An overview of the pathophysiology of MetS

The pathophysiology of MetS is complex, and many aspects remain unknown, but what has been clarified so far indicates a pivotal role for obesity and insulin resistance in its pathogenesis. Inflammation, adipokines, oxidative stress, chronic psychological stress, and changes in the composition of intestinal bacteria are also important players in the pathogenesis of MetS. Notably, there was an interesting relationship among these events, obesity, and insulin resistance. The binding of insulin to insulin receptors (IR) on the surfaces of tissues such as the liver, adipose tissue, and skeletal muscle, which can store glucose as glycogen or triglycerides, plays a very important role in regulating glucose and lipid metabolism in the human body. These receptors have tyrosine kinase properties and consist of four subunits. The binding of insulin to these receptors leads to the auto-phosphorylation of the receptors at specific tyrosine residues, which recruits molecules called IRSs (IRS1 and IRS2) and their phosphorylation. Phosphorylation of these adapter molecules makes them able to activate PI3K. The PI3K/AKT signaling pathway is involved in GLUT4 translocation to the cell membrane. GLUT4 can bring glucose into the cell (Yan et al., 2022). Therefore, the disturbances in the events mentioned above can cause insulin resistance, leading to disturbances in glucose and lipid metabolism. Insulin resistance in people with MetS can be caused by obesity or genetic factors. Among the genetic abnormalities associated with insulin resistance is a mutation in the insulin receptor gene, which, in a severe form, may cause death in childhood and, in a less severe form, causes marked insulin resistance and hyperinsulinemia. A mutation in peroxisome proliferator-activated receptor-γ (PPAR-γ), a transcription factor, is another genetic abnormality associated with insulin resistance. This transcription factor regulates the transcription of several genes involved in inflammation, insulin sensitivity, and adipocyte differentiation, and its mutations may be associated with hyperinsulinemia, T2D, and hypertension (Gallagher et al., 2010). Inactivating mutations in the IRS-1 and IRS-2 genes are among the genetic abnormalities associated with insulin resistance (Toyoshima et al., 2025). Obesity, which can be caused by various factors such as over-nutrition, lack of physical activity, genetic factors, aging, and changes in the gut microbiota, is a key contributor to insulin resistance and MetS (Abdullah et al., 2024; Ahmed et al., 2021).

In a study of 12,047 adults in the United States, the prevalence of MetS was 61.6% among obese individuals, 33.2% among overweight individuals, and only 8.6% among normal-weight individuals (Shi et al., 2020). Obesity causes changes in adipose tissue that are associated with tissue dysfunction and insulin resistance. Among these changes is adipocyte hypertrophy and subsequent hypoxia, which is considered a stressful condition for adipocytes and appears to lead to enhanced secretion of key chemokines and cytokines, such as MCP-1, TNF-α, RANTES, and M-CSF-1, by hypertrophied adipocytes. The secretion of these inflammatory mediators can strengthen the infiltration of immune cells, especially macrophages, into adipose tissue and the inflammatory response (Chan et al., 2017; Lionetti et al., 2009; Ye et al., 2022). In addition, the polarization of macrophages residing in adipose tissue is altered in obesity, and the pro-inflammatory phenotype (M1) can be enhanced (Chan et al., 2017). It should be noted that macrophages are classified into two types, M1 and M2, depending on the activation pathway (classical or alternative), which have pro-inflammatory and anti-inflammatory properties, respectively (Vaghari-Tabari et al., 2022). The secretion of compounds such as free fatty acids and TNF-α following adipocyte hypertrophy and hypoxia can facilitate a shift in macrophage polarization toward the M1 phenotype. By secreting high levels of pro-inflammatory cytokines, especially TNF-α and IL-6, inflammatory macrophages can exacerbate insulin resistance in adipose tissue through autocrine and paracrine signaling. Additionally, these pro-inflammatory cytokines play a key role in systemic insulin resistance via endocrine signaling (Chan et al., 2017). Several mechanisms have been proposed to explain how these cytokines promote insulin resistance. For example, TNF-α can induce phosphorylation of specific serine residues in IRS-1, thereby converting this molecule into an inhibitor of insulin receptor tyrosine kinase activity. TNF-α can also activate JNK and NF-κB. These transcription factors are involved in disrupting insulin signaling. IL-6 can also contribute to the attenuation of insulin signaling by inducing SOCS-3. Besides, TNF-α can increase circulating free fatty acid levels by enhancing hormone-sensitive lipase activity in adipose tissue. NF-κB, which is a pivotal transcription factor in inflammation, is necessary for the induction of lipolysis in adipose tissue by TNF-α. High levels of free fatty acids have a very destructive effect on insulin signaling and play an important role in the pathogenesis of MetS (Kahn et al., 2006; Laurencikiene et al., 2007; Tilg and Moschen, 2008; Yan et al., 2022).

In skeletal muscle, free fatty acids can disrupt insulin signaling, weaken GLUT-4 translocation to the cell surface, and decrease glucose uptake. In the liver, free fatty acids can also enhance gluconeogenesis and lipogenesis. Inside cells, fatty acids compete with glucose for oxidation, and increasing intracellular fatty acid levels can attenuate the activities of hexokinase II, phosphofructokinase, and pyruvate dehydrogenase. All these events can cause hyperglycemia and subsequently hyperinsulinemia (Fahed et al., 2022; Kahn et al., 2006). In addition, increasing fatty acid delivery or attenuating their metabolism can increase levels of fatty acid metabolites, such as ceramide and diacylglycerol (DAG), within cells. These metabolites can disrupt PI3K/AKT signaling, increase ROS production, induce endoplasmic reticulum stress, and trigger inflammation (Ahmed et al., 2021; Kahn et al., 2006). All these events can enhance insulin resistance. Additionally, TNF-α and IL-6 can enhance the activity of NADPH oxidase, a major source of ROS in cells, and increase superoxide anion production, thereby exacerbating oxidative stress (Manna and Jain, 2015; Vaghari-Tabari et al., 2021). Oxidative stress can impair insulin signaling by several mechanisms. For example, high levels of ROS can activate JNK, which disrupts insulin signaling by phosphorylating specific serine residues in IRS (Manna and Jain, 2015). Oxidative stress may also impair IRS-1 phosphorylation and PI3K/AKT signaling by enhancing NF-κB activity, a central player in inflammation (Ogihara et al., 2004). Therefore, there is a two-way relationship between oxidative stress and inflammation, and each can strengthen the other. It should be noted that several factors can be involved in enhancing oxidative stress in obese people, which have been well explained elsewhere (Manna and Jain, 2015). Overall, inflammation acts as a link between obesity and insulin resistance and plays a crucial role in the pathogenesis of MetS. Interestingly, in addition to the aforementioned events in adipose tissue, changes in the gut microbiota can also cause inflammation, thereby exacerbating insulin resistance. Obesity and a high-fat Western diet can alter the composition of intestinal bacteria and increase gut permeability (Velasquez, 2018). Among these changes are increases in the populations of Firmicutes, Proteobacteria, and Lactobacillus, and decreases in the populations of Bacteroidetes, Akkermansia, and *Faecalibacterium prausnitzii* (Pinart et al., 2021; Wang et al., 2020a). Bacteria such as Akkermansia and *Faecalibacterium prausnitzii* have beneficial effects in strengthening the intestinal mucosal barrier, preventing excessive bacterial penetration, and probably reducing their populations. A reduction in the abundance of these beneficial bacteria may impair intestinal barrier integrity and facilitate bacterial translocation (Yan et al., 2022).

Enhancing bacterial penetration and increasing the entry of certain bacterial products, especially LPS from Gram-negative bacteria, into the circulation can lead to low-grade systemic inflammation. It is worth noting that LPS can also cross the intestinal mucosa by chylomicrons. Circulating LPS penetrates tissues such as adipose tissue and liver. LPS bound with LPS binding protein can activate CD14 in the plasma membrane of macrophages, and finally, TLR4 signaling can be activated in macrophages. TLR4 signaling through the MyD88 activates the MAPK pathways) ERK1/2, P38, JNK) and IKKB, which leads to the activation of NFKB and AP-1 and ultimately increases the production of inflammatory mediators (e.g., TNF-α and IL-6). As mentioned above, these pro-inflammatory cytokines have destructive effects on insulin signaling. Studies on human samples have also clearly shown the link between LPS, inflammation, and insulin resistance. For example, it has been shown that LPS can markedly induce TNF-α secretion by human adipose tissue, and that increased circulating LPS levels, termed metabolic endotoxemia, are associated with insulin resistance and components of the MetS (Al-Lahham et al., 2018; Boulangé et al., 2016; Lassenius et al., 2011; Liang et al., 2013; Radilla-Vázquez et al., 2016; Velasquez, 2018). Obesity causes changes in the secretion of hormones produced by adipose tissue, such as leptin, adiponectin, and resistin, resulting in decreased adiponectin levels and increased leptin and resistin levels (Forny-Germano et al., 2018; Steppan et al., 2001). These changes play an important role in insulin resistance and the pathogenesis of MetS. For example, adiponectin can activate AMPK signaling by enhancing phosphorylation of AMPK α at Thr172, thereby exerting a protective effect against insulin resistance (Wang et al., 2019). Adiponectin may enhance PPAR-α activity in skeletal muscle through activation of AMPK and p38MAPK, leading to increased expression of PPAR-α target genes such as carnitine palmitoyltransferase 1 (CPT-1) and enhanced fatty acid oxidation (Yoon et al., 2006). It is worth noting that AMPK plays a very important role in regulating metabolism. In situations requiring energy, AMPK can enhance glucose uptake and fatty acid oxidation and attenuate the synthesis of fatty acids and glucose (Yan et al., 2022). Therefore, it is clear that reduced adiponectin secretion in obesity impairs insulin signaling and metabolic regulation. In addition, adiponectin has anti-inflammatory properties, and a study in patients with MetS clearly showed an inverse relationship between total adiponectin concentration and markers of oxidative stress and inflammation (TBARS and CRP) (Frühbeck et al., 2017). Therefore, it is clear that decreased levels of this hormone exacerbate low-grade systemic inflammation and thus have destructive effects on insulin signaling.

Leptin resistance and hyperleptinemia are important factors linking obesity to insulin resistance and MetS. Leptin resistance can attenuate leptin’s inhibitory effect on SREBP1. Increased expression of SREBP1 can enhance lipid accumulation in metabolically important tissues such as the liver and skeletal muscle. Additionally, leptin resistance can attenuate fatty acid oxidation. In addition, leptin has pro-inflammatory properties and can stimulate the Th1 response and enhance the expression of TNF-α and IL-6 in macrophages (Gómez et al., 2025; Obradovic et al., 2021; Patel et al., 2008; Rochlani et al., 2017). These events can lead to insulin resistance. Interestingly, elevated levels of bacterial LPS can also enhance leptin expression in adipose tissue and significantly increase plasma leptin levels (Berkowitz et al., 1998). In fact, metabolic endotoxemia may also contribute to the development of hyperleptinemia. In addition to the above-mentioned effects, leptin can also enhance the production of resistin, which can promote insulin resistance through mechanisms such as increasing PTEN expression, attenuating PI3K/AKT signaling, and promoting inflammation (Barnes and Miner, 2009; Gunturiz Albarracín and Forero Torres, 2020). All the above points underscore the importance of changes in adipokine levels in the pathogenesis of insulin resistance and MetS. Chronic psychological stress can also cause disturbances in the regulation of the hypothalamic-pituitary-adrenal axis and increase the levels of glucocorticoids. Glucocorticoids can attenuate AMPK signaling, enhance lipolysis in adipose tissue, thereby increasing circulating free fatty acid levels, and promote gluconeogenesis in the liver by increasing the expression of phosphoenol pyruvate carboxykinase and glucose-6-phosphatase. These events can lead to insulin resistance and hyperglycemia (Paredes and Ribeiro, 2014). Therefore, chronic stress and hypercortisolemia play an important role in the pathogenesis of MetS. In addition, some other disorders, such as polycystic ovary syndrome (PCOS), hyperthyroidism, and hypothyroidism, are also associated with insulin resistance (Ali, 2015; Ebrahimpour et al., 2018; Iwen et al., 2013), and there may be an interesting relationship between these disorders and the MetS. The increased release of fatty acids from adipose tissue mentioned above plays a central role in dyslipidemia, another important component of MetS. Insulin resistance, in turn, enhances lipolysis in adipose tissue and increases circulating free fatty acid levels. Strengthening of lipogenesis in the liver due to high levels of free fatty acids following enhanced lipolysis in adipose tissue, and increasing levels of triglycerides and cholesterol esters, leads to increased VLDL production and hypertriglyceridemia. In the presence of high levels of circulating VLDL, the transfer of triglycerides from VLDL to HDL by CETP is enhanced, and HDL clearance is intensified, leading to a reduction in HDL concentration (Fahed et al., 2022; Iqbal et al., 2018).

Hypertension is another component of MetS. Hypertension in MetS can result from various mechanisms, including oxidative stress, reduced NO production, attenuation of insulin-mediated vasodilation due to insulin resistance, and stimulation of the renin-angiotensin system (Fahed et al., 2022). Angiotensin II can also be produced by adipose tissue, and increased production of this key blood pressure regulator is associated with obesity and insulin resistance. Insulin resistance can also increase the expression of angiotensin II receptors. Angiotensin II, in addition to the vasoconstrictor effects and increased sodium reabsorption in the kidney, can also strengthen oxidative stress. All these effects are in line with increased blood pressure (Benigni et al., 2010; Katsimardou et al., 2020; Rochlani et al., 2017). Although what was reviewed above may seem extensive, it is only a brief overview of the pathogenesis of MetS, and targeting these key events can improve MetS. Interestingly, curcumin can affect all the events reviewed above, and its effects are also in line with improving MetS. In the next part, these effects will be reviewed in detail. Table 1. Major pathophysiological mechanisms linking adipose tissue dysfunction and insulin resistance to the major components of MetS.

**Table 1:** Major pathophysiological mechanisms linking adipose tissue dysfunction and insulin resistance to the principal components of MetS.

Pathophysiological alteration	Main molecular/metabolic mechanisms	Major downstream effects	MetS component(s)	references
Adipocyte hypertrophy and adipose tissue hypoxia	Increased secretion of MCP-1, TNF-α, IL-6, RANTES, and M-CSF-1; enhanced macrophage recruitment and polarization toward the pro-inflammatory M1 phenotype	Chronic low-grade inflammation and impairment of insulin signaling	Insulin resistance, hyperglycemia	(Chan et al., 2017; Lionetti et al., 2009; Ye et al., 2022)
Inflammation-mediated impairment of insulin signaling	TNF-α-induced serine phosphorylation of IRS-1 and activation of JNK/NF-κB; IL-6-mediated induction of SOCS3	Attenuation of insulin receptor signaling and promotion of insulin resistance	Insulin resistance, hyperglycemia	(Kahn et al., 2006; Laurencikiene et al., 2007)
Enhanced adipose tissue lipolysis and FFA release	Insulin resistance and inflammatory signaling increase lipolysis; TNF-α enhances hormone-sensitive lipase activity	Increased circulating free fatty acids and enhanced FFA delivery to liver and skeletal muscle	Insulin resistance, hyperglycemia, dyslipidemia	(Fahed et al., 2022; Laurencikiene et al., 2007)
Elevated FFA delivery to skeletal muscle	Impairment of insulin signaling and GLUT4 translocation; reduced glucose uptake and glucose oxidation	Peripheral insulin resistance and reduced glucose utilization	Hyperglycemia, hyperinsulinemia	(Fahed et al., 2022; Laurencikiene et al., 2007)
Elevated FFA delivery to the liver	Increased gluconeogenesis and lipogenesis; enhanced synthesis of triglycerides and cholesterol esters	Increased hepatic glucose output and VLDL production	Hyperglycemia, hypertriglyceridemia	(Fahed et al., 2022; Iqbal et al., 2018)
Increased hepatic VLDL production and altered HDL metabolism	Increased hepatic triglyceride availability promotes VLDL production; CETP-mediated transfer of triglycerides from VLDL to HDL accelerates HDL clearance	Hypertriglyceridemia and decreased HDL-C	Dyslipidemia	(Fahed et al., 2022; Iqbal et al., 2018)
Accumulation of intracellular lipid intermediates	Increased DAG and ceramide levels impair PI3K/AKT signaling and induce ROS production, ER stress, and inflammatory signaling	Further impairment of insulin signaling	Insulin resistance, hyperglycemia	Kahn et al. (2006)
Oxidative stress	Increased NADPH oxidase activity and ROS production; activation of JNK and NF-κB; impairment of IRS/PI3K/AKT signaling and reduced NO bioavailability	Insulin resistance and vascular/endothelial dysfunction	Hyperglycemia, hypertension	(Katsimardou et al., 2020; Ogihara et al., 2004)
Adipokine dysregulation	Reduced adiponectin/AMPK signaling; hyperleptinemia and leptin resistance; increased resistin-related signaling	Reduced fatty acid oxidation, increased ectopic lipid accumulation and inflammation, and impaired insulin sensitivity	Insulin resistance, dyslipidemia	(Barnes and Miner, 2009; Gunturiz Albarracín and Forero Torres, 2020)
Gut dysbiosis and metabolic endotoxemia	Altered gut microbiota and increased intestinal permeability enhance circulating LPS; activation of CD14/TLR4/MyD88 and downstream MAPK/NF-κB signaling increases inflammatory cytokine production	Systemic low-grade inflammation and worsening of insulin resistance	Insulin resistance, hyperglycemia	(Lassenius et al., 2011; Liang et al., 2013; Radilla-Vázquez et al., 2016)
Activation of the renin–angiotensin system	Increased angiotensin II production, enhanced angiotensin II receptor signaling, vasoconstriction, renal sodium retention, and increased oxidative stress	Increased vascular resistance and arterial blood pressure	Hypertension	(Benigni et al., 2010; Katsimardou et al., 2020; Rochlani et al., 2017)
Impaired insulin-mediated vasodilation and endothelial dysfunction	Insulin resistance reduces NO-dependent vasodilation; oxidative stress decreases NO bioavailability	Increased vascular tone and impaired vascular function	Hypertension	(Fahed et al., 2022; Katsimardou et al., 2020)

## 4 Curcumin and MetS: a natural compound with therapeutic application?

Given the prominent roles of oxidative stress and inflammation in the pathogenesis of T2D and cardiovascular disease, curcumin has attracted considerable attention for its potential to modulate cardiometabolic pathways and risk factors associated with these conditions. However, evidence demonstrating prevention of clinical diabetes or cardiovascular events remains limited. Although few statistics are available, an inverse relationship between turmeric consumption and cardiovascular mortality has been reported (Hashemian et al., 2019). Notably, one randomized controlled trial in individuals with prediabetes reported a lower incidence of progression to T2D following curcumin supplementation. Although this finding is clinically relevant, confirmation in independent, adequately powered trials is required before a preventive effect can be established (Chuengsamarn et al., 2012). Given that MetS significantly increases the risk of T2D and cardiovascular diseases, as mentioned in the introduction, studying the effects of curcumin on MetS has been a hot research topic in recent years. The mean daily intake of curcumin varies across societies depending on eating habits. For example, one of the studies on the Korean population reported values of 2.7-14.8 mg/day (Kwon, 2014), while this amount is much higher in India, and maybe between 2000 and 2500 mg/day (Fadus et al., 2017). Curcumin has generally been well tolerated in short-term clinical studies, including studies using relatively high oral doses. However, tolerability should not be equated with established long-term safety, and safety profiles may differ according to dose, duration, co-administered compounds, and formulation (Gupta et al., 2013b).

Although the relationship between the average daily intake of curcumin and the prevalence of MetS has not yet been studied, and statistics are not available, results from some meta-analyses of clinical studies in patients with MetS and related disorders clearly show that curcumin has beneficial effects on the components of MetS. These results have shown a significant relationship between curcumin intake and reductions in BMI, weight, waist circumference, insulin resistance index, and fasting glucose (Akbari et al., 2019; Tabrizi et al., 2018). These results have also shown that curcumin supplementation can help reduce triglycerides and total cholesterol (Tabrizi et al., 2018). Preclinical evidence for curcumin in MetS is summarized in Table 2. In this section, the effects of curcumin on the events involved in the pathogenesis of MetS that led to this beneficial outcome will be discussed in detail.

**Table 2:** Preclinical evidence on curcumin in MetS models.

Study	Model & Dose/Route	Formulation/Dose	Target/Endpoint	Key Result & Mechanism	Limitations/Notes
Weisberg et al. (Weisberg et al., 2008)	High-fat diet (HFD)-induced obese and ob/ob mice; dietary curcumin 3% w/w	Standard, 3% w/w	Glucose/insulin tolerance, adipose inflammation, hepatic NF-κB	Improved GTT/ITT and HbA1c; ↓ adipose macrophage infiltration; ↑ adiponectin; ↓ hepatic NF-κB—anti-inflammatory, insulin-sensitizing action.	Early study; high dietary dose.
Shao et al. (Shao et al., 2012)	C57BL/6J mice on HFD; 4 g/kg diet curcumin 2 days/week (oral)	Standard	Obesity/IR, hepatic lipogenesis, adipose inflammation	Prevented weight/fat gain and insulin resistance; ↓ SREBP-1c/ChREBP (hepatic lipogenesis); ↓ macrophage infiltration & inflammatory signaling in adipose.	Intermittent dosing.
Na et al., (Na et al., 2011)	HFD + streptozotocin diabetic rats; dietary curcumin 50–250 mg/kg/day	Dietary 50–250 mg/kg	Skeletal muscle insulin signaling (AMPK/LKB1), plasma glucose & lipids	Dose-dependently ↓ glucose & lipids; improved GTT/ITT; activated LKB1-AMPK, ↑ FA oxidation genes (CD36, CPT1); improved muscular insulin sensitivity.	Focus on muscle.
He et al., (He et al., 2012)	HFD mice; curcumin (oral; dietary)	Oral dietary	Oxidative stress signaling (Nrf2) & glucose intolerance	Curcumin corrected Nrf2 signaling defect in skeletal muscle, ↓ oxidative stress and improving glucose intolerance	Skeletal muscle focus.
Zhong et al. (Zhong et al., 2022)	HFD mice; oral curcumin	Oral	Gut microbiota & insulin sensitivity	↑ insulin sensitivity and metabolic parameters via gut microbiota modulation (restored microbial balance/SCFA profile).	Gut-liver axis emphasis.
Preez et al. (du Preez et al., 2019a)	Wistar rats with diet-induced MetS; low-dose nanocurcumin (oral)	Nano-curcumin, low-dose	Blood pressure, endothelial function, MetS score	Normalized hypertension and improved CV/endothelial indices at low doses; enhanced bioavailability formulation shows cardiometabolic benefit.	Bioavailability-enhanced.
Feng et al. (Feng et al., 2019a)	ApoE−/− mice	Oral	Hepatic steatosis, gut barrier & TLR4/NF-κB	Curcumin alleviated hepatic steatosis by strengthening intestinal barrier and dampening TLR4/NF-κB–mediated inflammation-gut-liver axis mechanism in MetS.	Gut-liver focus.
Hu et al. (Hu et al., 2013)	HFD-treated Sprague-Dawley rats; curcumin 200 mg/kg/day, oral gavage, 2 months	200 mg/kg/day	11β-HSD1 activity (rat & human intact cells); lipid/glucose profile	Improved plasma lipids; identified curcumin as a potent, selective 11β-HSD1 inhibitor, suggesting reduced local glucocorticoid activation as a mechanism in MetS.	Glucocorticoid link.
Kelany et al. (Kelany et al., 2017b)	High-fructose diet (HFD)-induced MetS in rats	Standard curcumin, oral	TNF-α, NF-κB, oxidative stress, MetS components	Improved MetS parameters; ↓ TNF-α, NF-κB activity, oxidative stress; attenuated insulin resistance and hypertension	Focus on fructose model
Pan et al. (Pan et al., 2017)	Male C57BL/6J obese mice (HFD; curcumin treatment 8 weeks, oral) & 3T3-L1 adipocytes	Oral	Body weight, fat mass, serum lipids, fasting glucose, insulin sensitivity, adipocyte differentiation markers	↓ body weight, fat mass, serum TG & cholesterol; ↓ fasting glucose; ↑ insulin sensitivity; stimulated lipolysis; upregulated ATGL, HSL, PPARγ/α, C/EBPα; in 3T3-L1 cells ↑ glucose uptake, ↓ glycerol release; overall improvement of glycolipid metabolism via PPARγ pathway	In vitro + in vivo.
Kobori et al. (Kobori et al., 2018a)	Obese mice (HFD)	Dietary curcumin	eIF2 signaling, ER stress, adipose tissue	mproved eIF2 signaling; ↓ lipid levels in white adipose tissue, reduced ER stress and inflammation	Adipose-specific effects
Ejaz et al. (Ejaz et al., 2009)	3T3-L1 cells (5–20 μM); HFD mice + curcumin 500 mg/kg diet, 12 weeks	500 mg/kg diet	Angiogenesis, adipogenesis, lipid metabolism	↓3T3-L1 differentiation & VEGF/VEGFR-2; apoptosis in adipocytes; ↓ body weight & fat; ↑AMPK, ↑CPT-1, ↓GPAT-1; ↓ PPARγ/C/EBPα; improved lipid profile	Angiogenesis angle.
Zou et al. (Zou et al., 2021)	C57BL/6 mice, HFD ± 0.2% Cur in diet for 10 weeks	0.2% in diet	Adiposity, insulin sensitivity, energy metabolism, FNDC5/irisin	Cur reduced adiposity, ↑ insulin sensitivity, ↑ plasma irisin, ↑ oxygen consumption & heat production, ↓ RER; activated FNDC5/p38 MAPK/ERK1/2 pathway in WAT & BAT (improved whole-body energy metabolism)	Browning/thermogenesis.
Maithilikarpagaselvi et al. (Maithilikarpagaselvi et al., 2016c)	Male Wistar rats; high-fructose diet for 10 weeks; curcumin oral treatment	Oral	Insulin signaling (IRS-1 phosphorylation), inflammatory markers (TNF-α, CRP, COX-2, PKCθ), oxidative stress (MDA, TOS, GPx), MAPKs (ERK1/2, p38)	Curcumin ↑ insulin sensitivity via IRS-1 modulation ↓ hyperinsulinemia, glucose intolerance & HOMA-IR, ↓ TNF-α/CRP/COX-2/PKCθ & prevented IκBα degradation, ↓ oxidative stress (MDA, TOS); ↑ GPx, ↓ ERK1/2-/p38; overall anti-inflammatory & antioxidant protection.	Serine kinase focus.
Cho et al. (Cho and Park, 2025)	Male C57BL/6 mice; HFD 60% ± curcumin 2.5 g/kg diet, 8 weeks	2.5 g/kg diet	Obesity, weight gain, glucose/lipid profile, adipocytes, DNA repair (pCHK1/pCHK2)	Curcumin ↓ weight gain, preserved glucose homeostasis, improved lipid profile; ↑ADMSC (Sca-1+ CD45^−^) and ↑pCHK1 in adipocytes; in human preadipocytes, ↓IL6/CCL2, ↑pCHK2 and ↓γH2AX under H2O2 stress; inhibited preadipocyte-to-adipocyte differentiation; mechanism: anti-inflammatory and enhanced DNA repair signaling	DNA damage repair mechanism.
Meléndez-Salcido et al. (Meléndez-Salcido et al., 2025)	5-week-old C57BL/6 mice; hypercaloric diets (HFD, high-fructose), 0.75% curcumin in diet for 16 weeks	0.75% in diet, 16 weeks	Kidney: inflammation (TNF-α), fibrosis, fatty acid metabolism (VLCAD, CPT1, PPARγ), injury (KIM1), morphology	Curcumin prevented TNF-α overexpression, ↓ CPT1 & KIM1, ↑ PPARγ expression, and ↓ fibrosis, lipid deposition, and morphological lesions induced by hypercaloric diets; shows renal protective, anti-inflammatory, anti-fibrotic and lipid-modulating effects	Renal protective effects
Ke et al. (Ke et al., 2025)	Male mice; HFD + curcumin 80 mg/kg/day, oral, 12 weeks	80 mg/kg/day	Pancreatic steatosis, insulin resistance, lipid metabolism (LATS2, FAS, YAP, SREBP2, p53)	Curcumin ↓ pancreatic fat infiltration and inflammation, ↑ insulin sensitivity; ↓ LATS2, FAS, YAP, SREBP2 and ↑ p53, suggesting modulation via YAP/p53 pathway	Pancreatic focus.
Ozturk et al. (Ozturk et al., 2025)	Female Sprague–Dawley rats; turmeric 80 mg/kg/day, oral gavage, 60 days; MetS induced by 20% fructose diet	Turmeric 80 mg/kg/day	Oxidative stress markers (MDA, SOD, CAT), lipid profile (TC, LDL, HDL), glucose, body weight, blood pressure	Turmeric ↓MDA, glucose, TC, LDL, body weight, and systolic BP; ↑ HDL, vitamin A/C/E, SOD, CAT; protective effect attributed to ↑ antioxidant defense and biochemical parameters	Turmeric extract.

### 4.1 Curcumin, obesity, insulin resistance, and glycemic control

Several studies on HFD-fed mice have shown that curcumin treatment can attenuate the effects of a high-fat diet on weight/fat gain (Shao et al., 2012; Um et al., 2013; Zou et al., 2021). Some studies on high-fat diet-fed mice have clearly shown that curcumin treatment can reduce adipocyte size in white adipose tissue and prevent high-fat diet-induced adipocyte hypertrophy (Islam et al., 2021; Li et al., 2021b). Another study showed that curcumin can inhibit adipocyte differentiation. It also seems that curcumin can activate caspases 3, 8, and 9 and induce apoptosis in preadipocytes. These events can attenuate adipogenesis (Wu et al., 2019). Studies have shown that curcumin inhibits the expression of adipogenic genes, including PPARγ and C/EBPα, and that its suppression of adipocyte differentiation may be due to this inhibitory effect (Kim et al., 2011; Wu et al., 2019). Several mechanisms have been proposed to account for curcumin’s inhibitory effects on adipogenic gene expression. For example, a study has shown that curcumin can increase TRAF4 m6A levels by attenuating ALKHB5 expression (an m6A demethylase), thereby enhancing TRAF4 translation. TRAF4 functions as an E3 ubiquitin ligase and enhances PPARγ degradation, thereby suppressing adipogenesis and preventing obesity (Chen et al., 2021). In addition to attenuating adipogenesis, curcumin may promote energy expenditure, thermogenesis, and browning of white adipose tissue (Song et al., 2018; Wang et al., 2015).

Curcumin can increase the expression of uncoupling protein 1 (UCP1), an important thermogenic protein, in brown adipose tissue, increase energy expenditure, and raise body temperature in response to cold (Song et al., 2018). Interestingly, a study in mice also showed that curcumin may promote the browning of inguinal white adipose tissue by increasing mitochondrial biogenesis and the expression of thermogenic genes such as Ucp1. The results of this study suggest that curcumin may exert this effect by increasing norepinephrine release and enhancing β3A receptor expression in inguinal adipose tissue (Wang et al., 2015). Curcumin has considerable anti-inflammatory effects and can attenuate low-grade systemic inflammation caused by obesity. Curcumin can attenuate MCP-1 secretion by adipocytes, reduce the expression of pro-inflammatory cytokines, including TNF-α and IL-6, in adipocytes, and reduce macrophage infiltration into adipose tissue (Gonzales and Orlando, 2008; Islam et al., 2021; Woo et al., 2007). Besides, curcumin has been shown to reduce serum RANTES levels (Bulboacă et al., 2019). However, the effect of curcumin on RANTES secretion by adipocytes in obesity needs to be clearly elucidated. In addition, curcumin appears to decrease the percentage of M1 macrophages and increase the percentage of M2 macrophages in white adipose tissue. Curcumin also affects cytokine secretion by immune cells infiltrating white adipose tissue, thereby reducing levels of some pro-inflammatory cytokines, such as IL-1β and TNF-α, while increasing IL-10 secretion, an anti-inflammatory cytokine (Jang et al., 2008). In addition, curcumin can attenuate certain signaling pathways that mediate the effects of TNF-α and IL-6 in impairing insulin signaling. For example, curcumin has an inhibitory effect on NF-κB p65 expression, JNK activity, and serine phosphorylation of IRS-1 in adipocytes (Priyanka et al., 2017; Wang et al., 2009). Additionally, it has been shown that curcumin can increase insulin-stimulated glucose uptake in adipocytes and synergistically enhance insulin-mediated GLUT4 translocation to the cell surface in myoblasts (Mohiti-Ardekani et al., 2019; Wang et al., 2009). Furthermore, curcumin can decrease the expression of STAT3 and SOCS3 and increase the expression of IRS-1 in skeletal muscle (Zaheri et al., 2019). Therefore, curcumin appears to both reduce the expression of inflammatory mediators and weaken their effects in promoting insulin resistance.

Curcumin can also attenuate the stimulatory effect of TNF-α on lipolysis in adipocytes. The results of a study suggest that this effect of curcumin is probably exerted through the suppression of ERK1/2 phosphorylation and the reversal of the reduction of perilipin expression in adipocytes (Xie et al., 2012). In addition, curcumin can weaken the effect of fatty acids on insulin resistance in skeletal muscle. The results of a study suggest that palmitate significantly decreases IRS-1 expression, whereas curcumin can reverse this reduction, enhance AKT phosphorylation, and improve glucose uptake in skeletal muscle cells (Deng et al., 2012). Tetrahydrocurcumin, a metabolite of curcumin, also showed similar effects in hepatocytes incubated with oleic acid (Chen et al., 2018). The results of a study on high-fat diet rats have also shown that treatment with curcumin at a dose of 80 mg/kg reduces circulating levels of TNF-α and free fatty acids, normalizes plasma insulin levels, and reduces the insulin resistance index (HOMA-IR) (El-Moselhy et al., 2011). These in vivo findings can confirm the aforementioned in vitro findings. Therefore, curcumin can both weaken the inflammatory effect on the release of fatty acids from adipose tissue and neutralize the effect of free fatty acids on enhancing insulin resistance. As mentioned in the previous section, high levels of free fatty acids can increase metabolite levels, such as DAGs and ceramide, which in turn trigger ER stress, oxidative stress, and inflammation, thereby strengthening insulin resistance. Curcumin can suppress glycerol-3-phosphate acyltransferase 1 and adipose triglyceride lipase, reduce the levels of some DAGs, and attenuate ER stress (Kobori et al., 2018b; Wang et al., 2016)—however, the relationship between curcumin’s inhibitory effects on them and insulin sensitivity warrants investigation. As mentioned above, oxidative stress is an important contributor to the disruption of insulin signaling and is related to inflammation. The results of a study clearly showed that curcumin can attenuate palmitate-induced ROS production in skeletal muscle cells (Sadeghi et al., 2018). Additionally, the study in diabetic rats showed that curcumin can reduce the expression of the p47 (phox) and gp91 (phox) subunits of NADPH oxidase 2 and lower hydrogen peroxide levels in the spinal cord (Zhao et al., 2014). However, the relationship between the effect of curcumin on NADPH oxidase-mediated oxidative stress and insulin sensitivity in metabolically important tissues such as the liver, adipose tissue, and skeletal muscle is unclear and needs to be investigated in further studies.

In a study conducted in fructose-fed rats, curcumin reduced oxidative stress markers, including total oxidant status (TOS) and MDA, and increased glutathione peroxidase activity in skeletal muscle. The results of this study also showed that curcumin treatment can reduce plasma levels of TNF-α and CRP and lower HOMA-IR values, indicating attenuation of insulin resistance (Maithilikarpagaselvi et al., 2016c). A study conducted on obese rats on a high-fat diet also reported similar results, showing that curcumin treatment reduced TOS and MDA levels in skeletal muscle and plasma insulin and glucose levels, and decreased HOMA-IR values (Maithilikarpagaselvi et al., 2016a). These results suggest that curcumin’s antioxidant and anti-inflammatory properties are important in attenuating insulin resistance. Some studies have suggested that curcumin’s antioxidant properties may be related to its enhancing effect on NRF2, a transcription factor involved in enhancing antioxidant defense. One of these studies has shown that curcumin may attenuate the effect of ROS in causing insulin resistance in hepatocytes through enhancing the nuclear translocation of NRF2 (Zhao et al., 2011). The results of a study on high-fat diet-fed mice have shown that a high-fat diet can decrease the total or nuclear levels of NRF2 and heme oxygenase-1, a downstream target of NRF2 in skeletal muscle, while curcumin treatment can markedly reverse these effects (He et al., 2012). Subsequent studies have suggested that these effects of curcumin may be due to inhibition of the destructive effects of inflammation on NRF2. For example, an interesting study showed that TNF-α, which was mentioned in the previous section for its destructive role in causing insulin resistance, can increase the expression of keap1 and increase the polyubiquitination of NRF2, leading to the inhibition of NRF2 signaling, while curcumin can inhibit these effects. In fact, curcumin seems to be able to neutralize the effects of inflammation in weakening the antioxidant defense and strengthening the oxidative stress, thereby increasing insulin sensitivity (Ren et al., 2019). As mentioned in the previous section, changes in the composition of intestinal bacteria and attenuation of gut barrier integrity are important events that can contribute to the development of insulin resistance and play a role in the pathogenesis of MetS. The importance of these events and their relationship with low-grade systemic inflammation were also discussed. curcumin can mitigate the adverse effects of a high-fat diet on the expression of key intestinal tight junction proteins and strengthen the integrity of the intestinal mucosal barrier (Huang et al., 2021).

Besides, in a study conducted on obese mice with a high-fat diet, it was shown that dietary curcumin can reduce the ratio of Firmicutes/Bacteroidetes, increase the population of Akkermansia (bacteria with anti-inflammatory properties), decrease the population of Desulfovibrio bacteria (endotoxin-producing bacteria), reduce circulating levels of LPS, and improve insulin sensitivity (Li et al., 2021b). Besides, a study conducted in mice suggests an inverse relationship between the *Akkermansia muciniphila* population and adiposity, inflammatory markers, and insulin resistance markers, indicating the importance of Akkermansia in normal metabolism (Schneeberger et al., 2015). These interesting results suggest that curcumin may reduce circulating LPS levels and attenuate metabolic endotoxemia by neutralizing the deleterious effects of obesity and a high-fat diet on intestinal bacterial composition, ultimately leading to reduced low-grade systemic inflammation and improved insulin sensitivity. Another recent study also reported relatively similar results, which further strengthens this hypothesis. The results of this study have shown that a high-fat diet can decrease the populations of Bacteroidetes and Bifidobacterium spp. and increase the populations of Enterobacterales and Firmicutes, while treatment with curcumin can reverse these effects. The results of this study also showed that curcumin can reduce the serum levels of LPS and TNF-α, and attenuate insulin resistance (Huang et al., 2021). As mentioned in the previous section, LPS can enhance NF-κB activation and the production of inflammatory mediators by entering metabolically active tissues and activating TLR4/MyD88 signaling in macrophages, ultimately strengthening insulin resistance. Curcumin suppresses the expression of TLR4, the phosphorylation of ERK, JNK, P38, and NF-κB, and the production of pro-inflammatory cytokines such as TNF-α and IL-6 in macrophages (Zhou et al., 2015). The results of a study on mice with a high-fat diet have also shown that curcumin, in addition to strengthening the intestinal mucosal barrier and reducing the circulating levels of LPS, can down-regulate TLR4 and MyD88, suppress NF-κB signaling in liver tissue, and decrease the hepatic expression and serum levels of TNF-α (Feng et al., 2019b). All these results suggest that curcumin can not only attenuate metabolic endotoxemia and enhance insulin sensitivity by strengthening the intestinal mucosal barrier, increasing the population of beneficial bacteria such as Akkermansia, and decreasing the population of LPS-producing bacteria, but also attenuate the effects of LPS on inflammation by attenuating TLR4 signaling. Further analysis of this hypothesis may be an interesting topic for future studies.

Various studies have shown that curcumin can reverse these changes and affect the related mechanisms that are involved in insulin resistance. A study conducted in obese diabetic mice showed that curcumin treatment can enhance adiponectin expression in white adipose tissue (Weisberg et al., 2008). Additionally, a study in rats with MetS showed that curcumin can increase serum adiponectin levels (Kapar and Ciftci, 2020). The results of a meta-analysis on 6 trials with a total of 652 cases have also shown that curcumin supplementation can significantly increase serum adiponectin levels (Clark et al., 2019). It was mentioned in the previous section that adiponectin can activate AMPK and thus have protective effects against insulin resistance. A study on diabetic mice has clearly shown that curcumin can increase the expression of AMPK in liver tissue (Jiménez-Flores et al., 2014). The effect of curcumin in inducing GLUT4 expression and enhancing insulin sensitivity in skeletal muscle is also mediated by strengthening AMPK and AKT signaling (Mohiti-Ardekani et al., 2019). The results of a study also suggest that curcumin may increase fatty acid and glucose oxidation in skeletal muscles and improve insulin sensitivity through strengthening AMPK signaling (Na et al., 2011). The results of another study also suggest that curcumin enhances glucose uptake in skeletal muscle through the activation of AMPK/p38MAPK signaling pathway (Kim et al., 2010). In the previous section, we noted that adiponectin can enhance fatty acid oxidation in skeletal muscle by activating AMPK and p38MAPK signaling. Some studies have suggested that high concentration of adiponectin and low levels of inflammatory markers (hs-CRP and IL-1Ra) can predict MetS resolution (Ahonen et al., 2012). Therefore, curcumin’s effects in increasing adiponectin levels may further reduce low-grade systemic inflammation and oxidative stress in patients with MetS, thereby strengthening insulin sensitivity and improving MetS.

It appears that curcumin can also reduce leptin levels. A study conducted in rats with HFD-induced MetS showed that curcumin supplementation at 200 mg/kg once daily for 8 weeks significantly reduced serum levels of leptin, TNF-α, glucose, and insulin and attenuated insulin resistance (Kelany et al., 2017a). Another study on high-fat diet fed hamsters also showed that curcumin can reduce serum leptin levels (Jang et al., 2008). The results of a meta-analysis of 18 randomized controlled trials (RCTs) involving 1604 cases also showed that curcumin can significantly reduce leptin levels in people with MetS (Akbari et al., 2019). Additionally, a study has shown that curcumin can suppress leptin release from LPS-treated adipocytes (Maithilikarpagaselvi et al., 2016a), suggesting its ability to attenuate LPS-induced leptin production in adipose tissue. In addition, it is possible that curcumin has beneficial effects in improving leptin signaling in obese individuals. curcumin can attenuate leptin resistance in obese rats (Azza et al., 2013; Hajavi et al., 2017). KBH-1, a herbal combination consisting of *Curcuma longa*, *Saururus chinensis*, and *Polygala tenuifolia*, also had beneficial effects on leptin signaling and on reducing leptin resistance in rats with HFD-induced obesity (Lee et al., 2016). The effect of leptin resistance in weakening the oxidation of fatty acids was also mentioned. These events, as mentioned in the previous section, can enhance insulin resistance. The results of a study suggested that curcumin can reduce lipid accumulation and increase insulin sensitivity in mice with HFD-induced obesity through attenuation of SREBPs pathway (Ding et al., 2016). However, the relationship between curcumin’s effects and its ability to weaken leptin resistance remains unclear. According to the pro-inflammatory properties of leptin mentioned in the previous section, curcumin’s effects in reducing leptin levels in obesity may be in line with further weakening low-grade systemic inflammation, which in turn may improve insulin sensitivity. curcumin can also attenuate the expression of resistin in adipocytes (Ying - and Ling -, 2010). Moreover, a study clearly showed that curcumin can reduce serum resistin levels in rats with MetS (Kapar and Ciftci, 2020).

In a study conducted in rats, it was shown that chronic mild stress can increase whole-blood free fatty acid levels and serum levels of glucose, insulin, leptin, triglycerides, LDL, and corticosterone, an important glucocorticoid. This study also showed that chronic mild stress can attenuate insulin sensitivity. Interestingly, this study showed that curcumin treatment can improve depressive-like behavior in these rats and reverse the above-mentioned changes (Shen et al., 2017). The results of a study on rats with HFD-induced obesity have also shown that curcumin is a competitive inhibitor of 11β-Hydroxysteroid dehydrogenase 1. In humans, this enzyme catalyzes the conversion of biologically inactive cortisone to biologically active cortisol. The results of this study also showed that curcumin treatment reduces serum levels of glucose, cholesterol, triglycerides, and LDL-C (Hu et al., 2013). However, more studies are needed to clarify the relationship between curcumin’s inhibitory effect on 11β-Hydroxysteroid dehydrogenase 1 and these beneficial effects. Additionally, curcumin can attenuate ACTH-mediated cortisol secretion from the adrenal gland (Enyeart et al., 2009). All these effects of curcumin may be in line with attenuating the effects of chronic psychological stress and hypercortisolemia in strengthening insulin resistance and the pathogenesis of MetS. In the previous section, it was noted that certain conditions, such as PCOS, are associated with insulin resistance and MetS. In a study in rats with PCOS, curcumin treatment reduced the insulin resistance index and serum levels of IL-6 and CRP (Mohammadi et al., 2017).

Recent studies in women with PCOS have reported improvements in selected glycemic and insulin-resistance-related outcomes; however, these findings are specific to PCOS populations and should not be directly generalized to MetS (Abdelazeem et al., 2022; Chien et al., 2021). Besides, it would be interesting to study the effects of curcumin on insulin resistance and MetS parameters in other conditions (e.g., hyperthyroidism and hypothyroidism) that may be associated with an increased risk of insulin resistance and MetS. In addition to enhancing insulin sensitivity, curcumin appears to stimulate insulin secretion from the pancreas in response to glucose, thereby ameliorating hyperglycemia. In a study conducted in INS-1 cells, curcumin was shown to increase insulin gene expression and enhance glucose-stimulated insulin secretion. The results of this study suggest that curcumin may attenuate the effects of high glucose on the expression and secretion of insulin from pancreatic β cells by strengthening activation of the PI3K/Akt/GLUT2 signaling pathway (Song et al., 2015). Another study also suggests that curcumin can stimulate the secretion of glucagon-like peptide-1 (GLP-1) from enteroendocrine L cells, thereby enhancing insulin secretion and improving glucose tolerance (Kato et al., 2017). Another study has shown that curcumin can directly inhibit GLUT-1 and has hypothesized that attenuation of glucose absorption via inhibition of this transporter in intestinal epithelial cells may be related to curcumin’s hypoglycemic effects (Gunnink et al., 2016). In addition, it appears that curcumin can attenuate gluconeogenesis in the liver, which may also be related to its hypoglycemic effects. Mechanistically, curcumin can enhance the induction of Phosphodiesterase 4B in the liver and attenuate cAMP/PKA signaling. Besides, curcumin can reduce the accumulation of acetyl-CoA by attenuating mitochondrial fatty acid oxidation, thereby maintaining the activity of pyruvate dehydrogenase, suppressing the activity of pyruvate carboxylase, and attenuating the shift of pyruvate from oxidation to gluconeogenesis. All of these events can attenuate gluconeogenesis in the liver (Wang et al., 2018). Overall, the review in this section shows that curcumin can not only attenuate adipogenesis in adipose tissue but also attenuate the adverse effects of obesity on insulin signaling and target most of the important related mechanisms.

These results suggest that curcumin may be a useful compound in improving insulin resistance and hyperglycemia in MetS (Fig. 2). Undoubtedly, future studies will further analyze this hypothesis and clarify additional dimensions of curcumin’s effects in improving obesity and insulin resistance.

**Fig. 2:**
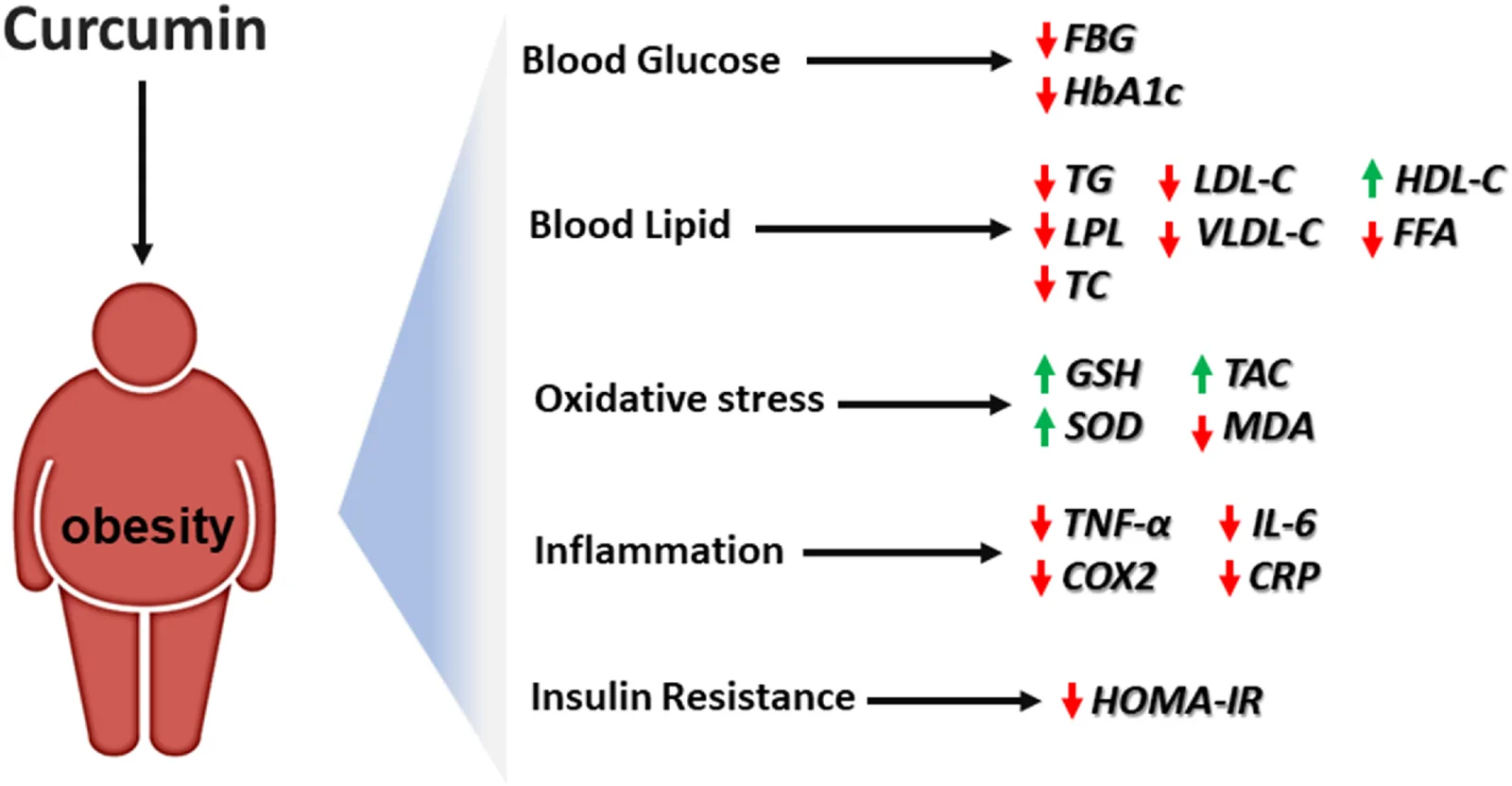
The therapeutic effects and potential mechanisms of curcumin on obesity. ↑ indicates increase and ↓ indicates decrease. Curcumin can lower blood glucose (FBG, HbA1c) and lipid levels (TG, LDL-C, VLDL-C, TC, LPL, FFA) while increasing HDL-C. Moreover, curcumin alleviates oxidative stress by decreasing MDA and enhancing antioxidant defenses (SOD, GSH, TAC). In addition, curcumin reduces inflammation by lowering TNF-α, COX2, IL-6, and CRP.

### 4.2 Curcumin and dyslipidemia

Several reports on animal models suggest that curcumin may help improve dyslipidemia. These studies have shown that curcumin can reduce serum triglyceride and increase HDL-C levels (Azhdari et al., 2019; Maithilikarpagaselvi et al., 2016a, 2016b). However, some meta-analyses of clinical studies have shown that curcumin, despite reducing triglyceride and total cholesterol levels, does not significantly increase HDL-C levels (Qin et al., 2017; Tabrizi et al., 2018). As mentioned in the previous section, a very important reason for dyslipidemia in MetS is increased delivery of free fatty acids from adipose tissue to the liver and enhanced VLDL production. In addition to these, based on what has been determined so far, curcumin’s effects in improving dyslipidemia may stem from its actions on key factors in lipogenesis and fatty acid oxidation, such as PPARα, SREBP, and AMPK. For example, studies in HFD-fed mice and high-cholesterol diet-fed LDLR(−/−) mice have shown that curcumin can enhance PPARα expression in the liver (Shin et al., 2011; Um et al., 2013; Zeng et al., 2017). PPARα plays an important role in lipid metabolism, enhancing β-oxidation of fatty acids in the liver and inhibiting VLDL production. In addition, PPARα can reduce triglyceride levels by increasing hepatic lipase expression, which hydrolyzes triglycerides in chylomicrons and VLDL, and by attenuating hepatic apoC-III expression, which inhibits plasma triglyceride clearance (Yoon, 2009). The effect of curcumin in increasing the expression of PPARα is related to the suppression of Notch-1 signaling in the liver, so the results of a study on HFD-fed rats showed that treatment with curcumin can reduce the expression of Notch-1 and Hes-1 in the liver. These effects are parallel to the increase in the expression of PPARα and other genes involved in fatty acid β-oxidation (Zhao et al., 2018).

The inhibitory effects of curcumin on SREBP1 were mentioned in the previous section. A study conducted on hepatocytes treated with oleic acid has also shown that curcumin can reduce the expression of SREBP-1c and fatty acid synthase (FAS), enhance AMPK phosphorylation, and reduce triglyceride and cholesterol levels. (Kang et al., 2013). These results may suggest that curcumin inhibits lipogenesis in the liver, even in the presence of high levels of fatty acids. Besides, the results of a study conducted on mice with HFD-induced obesity suggest that curcumin treatment can decrease the expression of SREBP1 and SREBP2 in the liver, which leads to the attenuation of the expression of their target genes, including genes related to cholesterol biosynthesis (e.g., 3-hydroxy-3-methylglutaryl coenzyme A (HMG-CoA) reductase) and genes related to the biosynthesis of fatty acids and triglycerides (e.g., FAS, Glycerol-3-phosphate acyltransferase, Acetyl-CoA carboxylase 1) (Ding et al., 2016). In the previous section, the effect of curcumin in enhancing GLP-1 secretion was mentioned. increasing GLP-1 levels can also decrease the expression of SREBP-1c, FAS, and stearoyl-CoA desaturase-1.Besides, exendin-4, a GLP-1 receptor agonist, can attenuate VLDL production in hepatocytes (Khound et al., 2017). However, it is not yet clear whether GLP-1 mediates curcumin’s effects on SREBP1 downregulation. SREBPs are transcription factors that play a central role in the biosynthesis of fatty acids, triglycerides, and cholesterol. It has been shown that the silencing of SREBP1c in bovine hepatocytes can attenuate the expression of acetyl-CoA carboxylase (a very important enzyme in the synthesis of lipids), increase the expression of enzymes involved in the oxidation of fatty acids (e.g., carnitine palmitoyl transferase І and II), and decrease the expression of genes involved in the assembly and transportation of VLDL (Apo B100, Apo E, MTTP) (Deng et al., 2014). Interestingly, some AMPK activators, including C24, have shown remarkable effects in attenuating the secretion of TG-rich VLDL from the liver of mice (Sun et al., 2021). Therefore, curcumin’s effects on AMPK activation may be an important contributor to its ability to improve dyslipidemia in MetS.

In addition to these, it appears that curcumin can enhance cytosine editing to uracil in apoB mRNA in the livers of rats by increasing the expression of catalytic polypeptide 1 (APOBEC-1), leading to reduced apoB100 levels (Qin et al., 2017), a major component of VLDL and LDL. All of these findings support observations from animal model studies indicating that curcumin reduces VLDL and LDL levels in MetS (Hu et al., 2013; Maithilikarpagaselvi et al., 2016a, 2016b). Moreover, curcumin can increase the levels and activity of LDL receptors (LDLRs) on the surface of hepatocytes by attenuating the expression of proprotein convertase subtilisin/kexin type 9 (PCSK9) (Qiu et al., 2023), thereby enhancing LDL uptake and reducing circulating LDL levels. It is worth noting that PCSK9 enhances the lysosomal degradation of LDL-R (Lagace, 2014). What was reviewed above shows that curcumin can not only attenuate lipolysis in adipose tissue and reduce the circulating levels of free fatty acids, but it is also able to attenuate the de novo synthesis of fatty acids and cholesterol, reduce the production of apoB100, and increases the levels of LDLR in the liver, which results in a reduction of plasma levels of triglycerides, total cholesterol, and LDL. Furthermore, curcumin can increase the excretion of cholesterol and triglycerides in stool. The results of a study on HFD-fed rats showed that curcumin can enhance the expression of cholesterol 7α-hydroxylase, a key enzyme in bile acid biosynthesis, in the liver, and significantly increase fecal cholesterol and triglycerides (Kim and Kim, 2010). Another study showed that turmeric supplementation in rats on a high-fat, high-cholesterol diet can increase serum total bile acid levels (Kim et al., 2012). These results suggest that curcumin may enhance cholesterol excretion by increasing bile acid synthesis in the liver. On the other hand, curcumin can increase the expression of apo AI, which is the main protein component of HDL, in the liver (Shin et al., 2011). Additionally, a study conducted in hamsters fed a high-fat diet clearly showed that curcumin treatment increased plasma levels of apo AI and HDL-C (Jang et al., 2008), suggesting that curcumin may enhance HDL production. In the previous section, it was noted that increasing triglyceride transfer from VLDL to HDL via CETP can increase HDL clearance and may help explain the decrease in HDL in MetS. The results of the study on high-cholesterol diet-fed rabbits showed that curcuminoids reduce CETP expression in liver tissue, increase plasma HDL levels, and decrease plasma TG, total cholesterol, and LDL levels (Elseweidy et al., 2015).

The results of a study on a high-cholesterol diet fed to LDLR(−/−) mice also showed that long-term curcumin treatment (18 weeks) can attenuate CETP activity in plasma, increase plasma HDL levels, and decrease plasma triglyceride, total cholesterol, and LDL levels (Shin et al., 2011). However, a clinical study in patients with MetS showed that curcumin supplementation at 500 mg twice daily for 6 weeks had no significant effect on CETP activity or serum HDL levels, although it significantly reduced serum triglyceride levels (Javandoost et al., 2018). Therefore, more clinical studies with longer treatment periods are needed to draw more accurate conclusions. In addition, the results of a study on rabbit adipocytes suggest that curcumin can enhance the efflux of cholesterol to apoA1, probably through the enhancement of a mechanism dependent on PPARγ/LXRα/ABCA1 (Dong et al., 2011), which indicates the effect of curcumin in enhancing the formation of HDL- 3 and the reverse cholesterol transport. Curcumin also affects the activity of paraoxonase 1. This enzyme enhances HDL’s ability to stimulate cholesterol efflux. Paraoxonase 1 can also hydrolyze oxidized LDL, which is an important atherogenic factor (Efrat and Aviram, 2010; Ikhlef et al., 2017; Shunmoogam et al., 2018). A study has shown that curcumin can enhance the expression of paraoxonase 1 in hepatocytes. However, this study did not show a similar effect in vivo (Schrader et al., 2011). In a study conducted in diabetic rats, curcumin treatment increased plasma paraoxonase 1 activity (Roxo et al., 2019). Another study showed that curcumin treatment can increase plasma paraoxonase 1 activity and reduce LDL oxidation susceptibility in rats (Singh and Ibrahim Rizvi, 2015). These preclinical findings support potential anti-atherogenic mechanisms of curcumin. However, whether these effects translate into a reduction in cardiovascular events in humans remains uncertain and requires confirmation in long-term clinical outcome trials. On the other hand, curcumin stimulates SR-B1 expression in macrophages (Sharifi-Rad et al., 2020); however, its effect on hepatic SR-B1 expression remains unclear. Since these receptors are involved in HDL breakdown in the liver, the results of some studies, especially clinical studies, showing that curcumin is ineffective at increasing HDL levels may be justified. To clarify this issue, further studies are needed to investigate curcumin’s effect on SR-B1 expression in the liver and its relationship to plasma HDL levels. Overall, the brief review in this section indicates that curcumin affects key mechanisms involved in the biosynthesis and metabolism of VLDL, HDL, and LDL, and may be a useful natural compound for improving dyslipidemia in MetS (Fig. 3). Undoubtedly, future studies will provide more information regarding the lipid-lowering effects of curcumin.

**Fig. 3:**
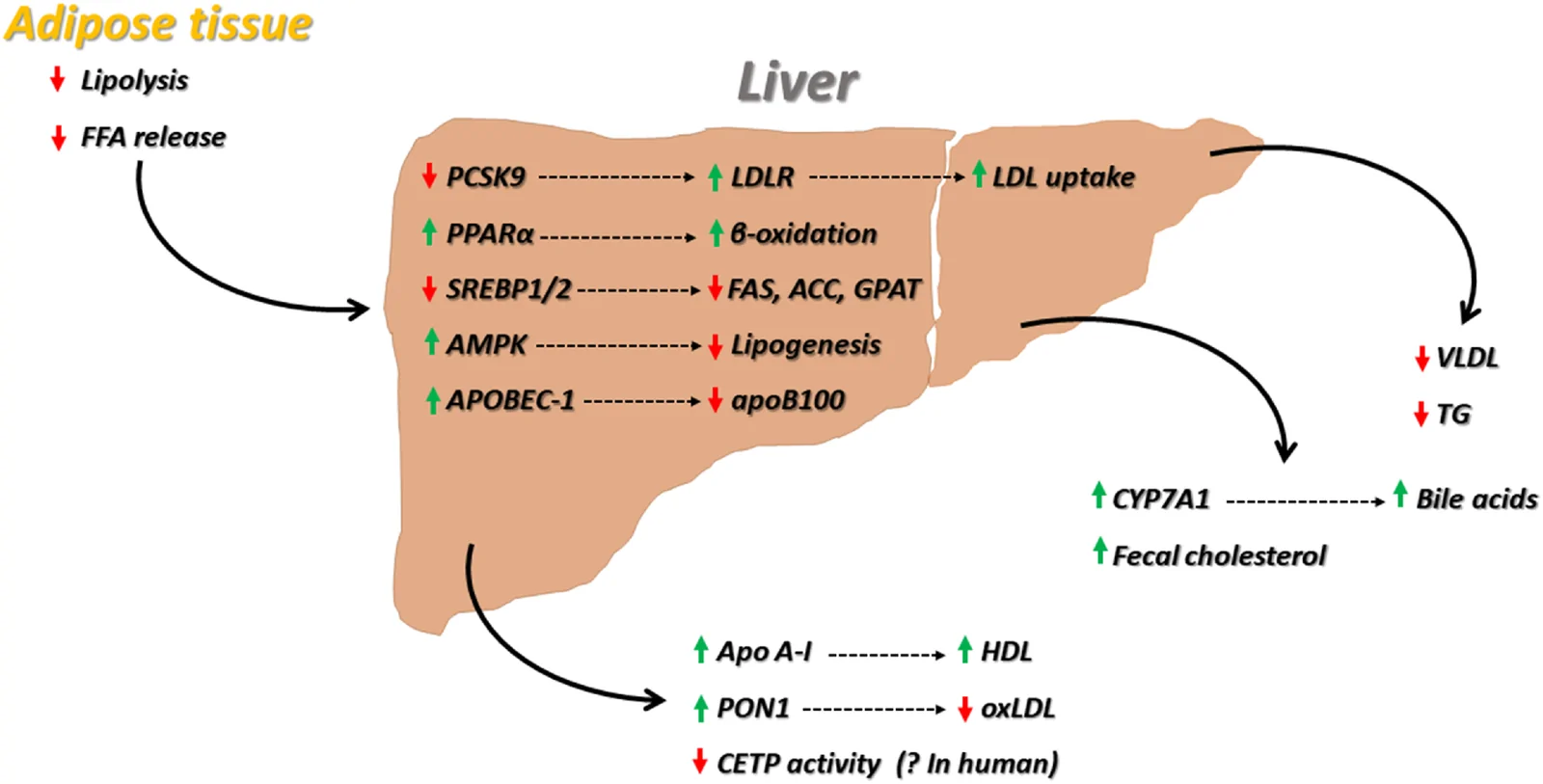
The regulatory effects of curcumin on lipid metabolism in MetS. Curcumin reduces lipolysis and FFA release, suppresses VLDL and TG production, and enhances LDL uptake by upregulating LDLR. It also promotes β-oxidation via PPARα, inhibits lipogenesis through SREBP and AMPK modulation, increases bile acid synthesis and fecal cholesterol excretion, and elevates ApoA-I, HDL, and PON1 activity.

### 4.3 Curcumin and hypertension

Studies in MetS animal models have shown that curcumin can normalize elevated systolic blood pressure (du Preez et al., 2019b; Kelany et al., 2017a). The mechanisms underlying curcumin’s effects in improving hypertension have not been widely studied, and findings are limited. These limited findings mostly indicate the importance of curcumin’s antioxidant properties in improving hypertension. For example, the results of a study on hypertensive rats suggest that curcumin can have beneficial effects on vascular function and attenuate blood pressure by enhancing the expression of eNOS in aortic tissue, increasing plasma nitrate/nitrite, reducing vascular superoxide anion production, attenuating oxidative stress, and strengthening blood reduced glutathione (GSH) (Nakmareong et al., 2011). Similar effects have been reported for tetrahydrocurcumin in hypertensive rats, and this metabolite of curcumin may be useful in improving hypertension due to these effects (Nakmareong et al., 2012). The results of a study conducted in rats with cadmium-induced hypertension also suggest that a significant part of the effects of tetrahydrocurcumin in improving hypertension may be due to its effects on increasing eNOS expression, decreasing iNOS expression, increasing nitrate/nitrite levels, attenuating oxidative stress, and increasing glutathione levels (Sangartit et al., 2014). Another study investigating the protective effects of curcumin in mice with cadmium-induced hypertension reported similar results (Kukongviriyapan et al., 2014). Another study on hypertensive rats suggested that curcumin’s antihypertensive effects may be due to its ability to enhance nitric oxide bioavailability and attenuate oxidative stress. The results of this study clearly showed that curcumin can downregulate p47phox NADPH oxidase in vascular tissue, reduce superoxide anion production, enhance eNOS expression, and increase plasma nitrate/nitrite (Boonla et al., 2014). Interestingly, curcumin can also attenuate the effect of angiotensin II in inducing oxidative stress by down-regulating the p47phox subunit of NADPH oxidase (Li et al., 2017). Previously, we mentioned the production of angiotensin II, a key regulator of blood pressure, from adipose tissue and the relationship between its increased production and obesity and insulin resistance. Additionally, the effect of insulin resistance on increasing the expression of angiotensin II receptors was mentioned.

Although the effects of curcumin on the production of angiotensin II in adipose tissue in obesity are not clear yet, the effect of curcumin in reducing the concentration of angiotensin II in the brain tissue of rats has been reported (Kim et al., 2019). Additionally, curcumin has been shown to reduce angiotensin-converting enzyme activity in the plasma of hypertensive rats (Boonla et al., 2014). In addition, a study clearly showed that curcumin can downregulate angiotensin II receptor type 1 (AT1R) in vascular smooth muscle cells by attenuating the binding of specificity protein 1 (SP1) to the AT1R gene promoter. The in vivo results of this study also showed that treatment with curcumin improves hypertension induced by angiotensin II in rats, and this effect is probably due to the reduction of ATR1 expression in arteries and the weakening of the vasoconstriction effects of angiotensin II (Yao et al., 2016). In fact, these results suggest that curcumin’s inhibitory effect on ATR1 expression in vascular smooth muscle cells is also related to its effects in improving hypertension. The results of another study also showed that curcumin attenuates the effect of angiotensin II in inducing the migration of vascular smooth muscle cells, which plays an important role in the pathogenesis of hypertension. The results of this study have suggested that this effect of curcumin may be due to its effects in attenuating NFKB activity and suppressing NFKB-mediated NLRP3 expression in vascular smooth muscle cells (Han et al., 2019). A study on (NLRP3−/−) mice has clearly shown that the lack of NLRP3 attenuates the effects of angiotensin II in inducing hypertension (Ren et al., 2017). Investigating the relationship between the above-mentioned findings and curcumin’s effects on ATR1, as well as its antioxidant effects, could be helpful. The results of a study also suggest that curcumin reduces blood pressure in rats by attenuating components of the brain renin-angiotensin system. The results of this study showed that curcumin can reduce ACE concentration and activity, angiotensin II concentration, and aldosterone concentration in the brain tissue of mice. However, the reduction of aldosterone was not statistically significant (Kim et al., 2019). It should be noted that the brain renin-angiotensin system appears to play an important role in the development and maintenance of hypertension (Marc and Llorens-Cortes, 2011). Another mechanism recently suggested to underlie curcumin’s effects in improving hypertension is changes in the composition of intestinal bacteria and an increase in the population of butyrate-producing bacteria. The results of a recently published clinical study of obese or overweight adults recovered from cancer clearly show that increased serum and fecal levels of butyrate are associated with lower blood pressure (Tilves et al., 2022).

A study conducted in hypertensive rats suggests that curcumin can increase plasma butyrate levels, which may be related to an increase in the population of butyrate-producing bacteria in the gut. The results of this study also showed that curcumin treatment can reduce blood pressure in hypertensive rats (Li et al., 2021a). In addition, curcumin can reduce aortic pulse wave velocity (aPWV) in old mice by reducing collagen and AGE levels, indicating improved aortic stiffness (Fleenor et al., 2013). The results of a study conducted in young obese men also suggest that curcumin can improve aortic stiffness, and this effect may be related to higher levels of IL-13 (Campbell et al., 2017). It should be noted that aortic stiffness predicts the development of hypertension in non-hypertensive individuals (Vaghari-Tabari et al., 2021) and that PWV is a non-invasive gold-standard method for assessing arterial stiffness (Gurovich and Braith, 2011). In overall, based on the studies that have been conducted so far, several major mechanisms for the antihypertensive effects of curcumin are suggested, including up-regulating eNOS, weakening oxidative stress, strengthening anti-oxidant defense, reducing the expression of ATR1 in arteries, attenuating the effects of angiotensin II in increasing the migration of vascular smooth muscle cells, attenuating brain renin-angiotensin system, and increasing the population of butyrate-producing bacteria in the gut. However, for more accurate conclusions, more studies on MetS animal models are needed.

Taken together, these mechanistic findings suggest that the actions attributed to curcumin should not be viewed as a series of independent molecular effects. Inflammation, oxidative stress, adipokine imbalance, altered lipid flux, insulin resistance, gut-barrier dysfunction, and endothelial abnormalities form an interconnected pathophysiological network in MetS. Modulation of upstream nodes such as NF-κB, NRF2, AMPK, PPARs, and PI3K/AKT may therefore influence several downstream metabolic phenotypes simultaneously. However, the strength of evidence for these pathways is uneven: many mechanistic relationships are derived predominantly from cellular or animal models, whereas confirmation in human MetS remains limited. Accordingly, mechanistic breadth should be interpreted as biological plausibility rather than evidence of broad clinical efficacy. The following section therefore evaluates separately the extent to which these mechanistic predictions are supported by human intervention studies.

## 5 Clinical evidence regarding curcumin effects in improving MetS

In this section, we have focused on clinical evidence and briefly reviewed clinical studies investigating the effects of curcumin on components of MetS. One of these clinical studies conducted on overweight patients with 44 MetS who lost less than 2% of their body weight after 30 days of dietary and lifestyle modification. The results of this study showed that 30 days of supplementation with the complex of *Curcuma longa* extract (containing 95% curcumin) at a dose of 800 mg/dose/diet and sunflower phospholipids (containing 20% phosphatidylserine) mixed with 8 mg/dose/diet of piperine can increase weight loss from 1.88% to 4.91%. Additionally, the results of this study showed that this supplement can increase body fat and waistline reductions by 0.7% to 8.43% and 2.36% to 4.14%, respectively. In addition, the reductions in BMI and hip circumference increased from 2.1% to 6.43% and from 0.74% to 2.51%, respectively (Di Pierro et al., 2015). In another clinical trial conducted in patients with MetS, 59 patients were treated with a curcumin supplement at a dose of 1 g/day for 8 weeks, and the effect of this supplement on cytokine levels was compared with the placebo group. The results showed that curcumin supplementation significantly reduced serum MCP-1 and TNF-α levels, and this reduction remained statistically significant even after adjusting for potential confounders (Panahi et al., 2016a).

In a study conducted on overweight women, it was also shown that curcumin supplementation at a dose of 500 mg/day (two 250 mg capsules per day) for 8 weeks has a significant effect in reducing CRP serum levels (Dolati et al., 2020), indicating the possible effects of curcumin in attenuation of inflammation in overweight/obese individuals. Additionally, in a clinical trial conducted in patients with MetS, co-supplementation with curcuminoids (1 g/day) and piperine (10 mg/day) over eight weeks resulted in a significant reduction in serum CRP levels (Panahi et al., 2015). Serum LPS levels were significantly reduced by curcumin supplementation at 500 mg/day for 15 to 30 days in a clinical trial in patients with T2D (Yang et al., 2015). This effect may be attributed to curcumin’s capacity to modify the composition of gut bacteria. The effect of curcumin on the gut microbiota in individuals with MetS or T2D remains inadequately elucidated and requires further investigation. Although one clinical trial suggests that curcumin supplementation at a dose of 1 g/day for 6 weeks does not significantly improve serum pro-oxidant-antioxidant balance (PAB) in patients with MetS (Ghazimoradi et al., 2017), another clinical trial suggests that curcumin, in the form of nano-micelle, can be beneficial at a dosage of 80 mg/day for 12 weeks. This second study demonstrates that it decreases serum malondialdehyde (MDA) levels and increases total antioxidant capacity (TAC) in patients with MetS (Bateni et al., 2022; Ghazimoradi et al., 2017).

In patients with MetS, a clinical trial showed that a combination of curcuminoids (1 g/day) and piperine (10 mg/day) reduced serum MDA levels and increased superoxide dismutase activity after 8 weeks of supplementation (Panahi et al., 2015). A clinical trial in patients with MetS demonstrated that curcumin supplementation at 1000 mg/day for 8 weeks significantly increased serum adiponectin levels and decreased serum leptin levels (Panahi et al., 2016b). In a study of overweight individuals with suboptimal fasting plasma glucose levels, it was discovered that the administration of phytosomal curcumin at a daily dosage of 800 mg (which includes 200 mg of curcumin, 480 mg of phosphatidylcholine, 120 mg of phosphatidylserine, and 8 mg of piperine) for 56 days can significantly reduce serum cortisol levels (Cicero et al., 2020). It is reasonable to expect that curcumin may have beneficial effects in reducing insulin resistance associated with MetS. The results of a clinical trial involving patients with MetS demonstrated that curcumin supplementation, administered as nanocapsules at 80 mg per day for 2 months, significantly reduced fasting plasma glucose levels and the insulin resistance index (HOMA-IR) (Kheiripour et al., 2021).

The results of an additional clinical trial demonstrated that administering curcumin in a nanomicelle formulation at a daily dose of 80 mg for 12 weeks did not substantially improve insulin resistance or hyperglycemia (Bateni et al., 2021). Variations in the nano-formulation may be responsible for this discrepancy. Another study has suggested that overweight individuals with suboptimal fasting glucose levels may experience reductions in fasting plasma glucose and HOMA-IR after 56 days of supplementation with phytosomal curcumin at 800 mg per day (200 mg curcumin) (Cicero et al., 2020). The findings of a meta-analysis encompassing 26 clinical trials in individuals with MetS and related disorders suggest that curcumin consumption may confer benefits. In particular, it has been shown to decrease fasting glucose levels, HbA1c, and insulin resistance (HOMA-IR) (Tabrizi et al., 2018). A clinical trial in patients with PCOS suggested that curcumin supplementation at 500 mg per day for 12 weeks may improve fasting plasma glucose levels and reduce insulin resistance (Jamilian et al., 2020).

It is crucial to emphasize that most studies included patients without high blood pressure at baseline. It is imperative to conduct clinical studies involving patients with high blood pressure to draw valid conclusions. For example, a study of 50 patients with MetS found that administering curcumin in a nanomicelle formulation at 80 mg per day for 12 weeks did not yield any substantial changes in blood pressure (Bateni et al., 2021). The results of an additional clinical trial demonstrated that administering curcumin in nano-capsule form at a dose of 80 mg per day for 60 days did not have a substantial impact on systolic and diastolic blood pressure in patients with MetS (Kheiripour et al., 2021). A 56-day course of phytosomal curcumin supplementation at a dose of 800 mg per day, which included 200 mg of curcumin, significantly reduced systolic blood pressure in overweight subjects with suboptimal fasting plasma glucose levels, according to a study. The baseline was 131 ± 8 mmHg, and the measurements decreased to 126 ± 6 mmHg by the end of the study (Cicero et al., 2020). In the preceding section, we examined the potential advantages of curcumin in improving aortic stiffness and the correlation between aortic stiffness and hypertension. A clinical trial involving patients with MetS demonstrated that daily curcumin supplementation at 500 mg for 12 weeks significantly improved pulse wave velocity (PWV). The significance of this improvement was maintained even after accounting for confounding variables (Alidadi et al., 2021).

Qiu et al. (2023) conducted a systematic review and meta-analysis of RCTs and found that curcumin supplementation significantly improved several key parameters of MetS. Reductions in waist circumference (mean difference = −2.16 cm), fasting blood sugar levels (mean difference = −8.6 mg/dL), diastolic blood pressure, and inflammatory markers such as TNF-α and CRP were observed. Moreover, there were decreases in malondialdehyde (MDA), an indicator of oxidative stress, alongside increases in high-density lipoprotein cholesterol (HDL-C). The data suggest a potential role for curcumin in regulating metabolic, inflammatory, and oxidative stress pathways in patients with MetS (Qiu et al., 2023). The extensive meta-analyses that were conducted in 2025 provide additional support. Moradi Baniasadi et al. conducted a GRADE-assessed systematic review and dose-response meta-analysis that concentrated on anthropometric indices in individuals with T2D and prediabetes. The evidence for various outcomes was of moderate confidence, and they reported significant reductions in body weight, waist circumference, fat mass percentage, and hip circumference following turmeric/curcumin supplementation. Kehinde et al. conducted one of the most comprehensive syntheses to date, analyzing 104 randomized controlled trials (RCTs) and documenting significant enhancements in glycaemic control (including fasting blood glucose and HbA1c), lipid profiles (with decreases in triglycerides and LDL-C and increases in HDL-C), and reductions in systemic inflammation (evidenced by lower levels of CRP, TNF-α, and IL-6) (Kehinde et al., 2025; Moradi Baniasadi et al., 2025). In studies addressing curcumin’s poor bioavailability, nano-based and novel formulations have demonstrated potential. In patients with MetS, nano-micelle curcumin (80 mg/day for 8–12 weeks) significantly enhanced glycaemic parameters, lipid profiles (particularly triglycerides), oxidative stress levels, inflammation (e.g., NF-κB), and adiponectin levels, as demonstrated by Bateni et al. (2021, 2022). Wilar et al. (2025) similarly reported that nanocurcumin supplementation had broad-spectrum beneficial effects on anthropometric measures, glycaemic control, lipid levels, and blood pressure in patients with MetS. Nano/phytosomal curcumin may address the limitations of standard extracts, as formulation-specific effects underscore the importance of delivery systems in translating preclinical promise into tangible clinical benefits (Bateni et al., 2022; Wilar et al., 2025). Table 3. Human clinical evidence on the effects of curcumin on MetS and related diabetes- and cardiovascular-risk outcomes.

**Table 3:** Human clinical evidence on the effects of curcumin on MetS and related diabetes- and cardiovascular-risk outcomes.

Study	Population/design	Intervention	Duration	Main outcomes	Main findings	Trial registration/Identifier	**Safety/adverse events**	Key limitation
Chuengsamarn et al., 2012 (Chuengsamarn et al., 2012)	240 adults with prediabetes; randomized, double-blind, placebo-controlled trial	Curcumin extract 1500 mg/day vs placebo	9 months	Progression to T2D, FPG, OGTT, HbA1c, HOMA-β, HOMA-IR, adiponectin	0% progressed to T2D in curcumin group vs 16.4% in placebo; improved β-cell function, HOMA-IR and adiponectin	ClinicalTrials.gov: NCT01052025*	No serious adverse effects reported. Minor events in the curcumin group included itching (n = 1), constipation (n = 2), and vertigo (n = 1); no hypoglycemia was reported. Liver and renal safety indices were not significantly different from placebo.	Prediabetes rather than established MetS; registered protocol specified a planned 12-month intervention whereas the published trial reports 9 months
Di Pierro et al., 2015 (Di Pierro et al., 2015)	44 overweight individuals with MetS; randomized controlled preliminary trial	Bioavailable curcumin–phosphatidylserine complex vs phosphatidylserine	30 days after lifestyle run-in	Weight, BMI, body fat, WC, hip circumference	Greater reductions in weight, BMI, body fat and WC	No verified ClinicalTrials.gov NCT identified	Very good tolerability reported; no treatment-related dropout occurred. Specific adverse-event counts were not reported.	Small sample; very short treatment; preliminary trial
Panahi et al., 2015; Panahi et al., 2016b, Panahi et al., 2016a (Panahi et al., 2016a, 2016b)	117 subjects with MetS; randomized, double-blind, placebo-controlled trial; subsequent analyses of same cohort	Curcuminoids 1000 mg/day + piperine 10 mg/day vs placebo	8 weeks	CRP, MDA, SOD, TNF-α, IL-6, TGF-β, MCP-1, adiponectin, leptin	↓ CRP, MDA and inflammatory cytokines; ↑ SOD and adiponectin; ↓ leptin	No verified ClinicalTrials.gov NCT identified for this specific RCT	No serious adverse events reported. Mild events included diarrhea (n = 2), constipation (n = 2), headache (n = 1), and skin rash (n = 2) in the curcumin group; headache (n = 2) and constipation (n = 1) occurred in placebo recipients.	Several publications originate from the same RCT and should not be counted as independent trials
Yang et al., 2015 (Yang et al., 2015)	14 patients with T2D; short-term uncontrolled/self-comparison clinical intervention	Curcumin 500 mg/day	15–30 days	Albuminuria, Nrf2, oxidative and inflammatory parameters, LPS	↓ urinary albumin excretion; enhanced Nrf2-related antioxidant signaling and reduced inflammatory/LPS-related markers	Not identified	Adverse events were not adequately reported in the publication; therefore, safety cannot be reliably assessed from this study.	Very small sample; uncontrolled/self-comparison design; short duration; T2D rather than MetS-specific population
Ghazimoradi et al., 2017 (Ghazimoradi et al., 2017)	120 patients with MetS; randomized, double-blind, placebo-controlled, three-arm trial	Curcumin 1 g/day; phospholipidated curcumin 1 g/day; placebo	6 weeks	Pro-oxidant–antioxidant balance (PAB)	No convincing improvement in PAB with curcumin formulations	No verified ClinicalTrials.gov NCT identified	One case of nausea was reported in the phospholipidated-curcumin group; one case of abdominal pain occurred in the placebo group. No major safety signal was reported.	Short duration; primarily a biomarker study
Dolati et al., 2020 (Dolati et al., 2020)	40 sedentary overweight women; randomized, double-blind, placebo-controlled four-arm trial	Curcumin 500 mg/day, with/without aerobic training	8 weeks	Anthropometry, FPG, insulin, HOMA-IR, lipids, CRP	Improvements in selected anthropometric and metabolic indices; combination with exercise produced broader changes	No verified ClinicalTrials.gov NCT identified	No participant reported adverse side effects associated with curcumin ingestion.	Only ∼10 subjects/arm; interaction with exercise; population did not have established MetS
Cicero et al., 2020 (Cicero et al., 2020)	80 overweight subjects with suboptimal fasting glucose; randomized double-blind placebo-controlled trial	Phytosomal preparation 800 mg/day containing 200 mg curcumin, 480 mg phosphatidylcholine, 120 mg phosphatidylserine and 8 mg piperine	56 days	FPG/FPI, HOMA-IR, WC, BP, TG, HDL-C, cortisol, liver indices	Improved several metabolic parameters, particularly FPI, TG, selected liver indices and cortisol	No verified ClinicalTrials.gov NCT identified	No clinically significant adverse events were reported; three participants in the curcumin group reported abdominal discomfort. All participants completed the trial.	Population did not necessarily have established MetS; formulation-specific findings
Kheiripour et al., 2021 (Kheiripour et al., 2021)	30 patients with MetS; single-arm intervention	Nano-curcumin 80 mg/day	60 days	FPG, insulin, HOMA-IR, afamin	↓ FPG, insulin, HOMA-IR and afamin	No verified ClinicalTrials.gov NCT identified	Adverse events were not reported; safety assessment was insufficiently described.	No placebo/control group; small sample; safety reporting incomplete
Bateni et al., 2021 (Bateni et al., 2021)	50 patients with MetS; randomized, double-blind, placebo-controlled trial	Nano-micelle curcumin 80 mg/day vs placebo	12 weeks	FBS, HbA1c, HOMA-IR, lipids, BP, anthropometry	Significant benefit mainly for TG; most glycemic, anthropometric and BP comparisons vs placebo were not significant	ClinicalTrials.gov: NCT03534024	Detailed adverse-event results were not reported in the publication; safety reporting was therefore limited.	Small sample; several neutral outcomes; incomplete safety reporting
Bateni et al., 2022 (Bateni et al., 2022)	50 patients with MetS; randomized, double-blind, placebo-controlled trial; 43 completed	Nano-micelle curcumin 80 mg/day vs placebo	12 weeks	MDA, TAC, hs-CRP, adiponectin, NF-κB	MDA and adiponectin improved within curcumin group; several between-group effects were not significant	ClinicalTrials.gov: NCT03514667	No serious adverse effects were reported during the intervention. Seven participants discontinued overall (3 curcumin; 4 placebo), with no significant difference in dropout rates.	Small sample; several effects significant only within group rather than between groups
Alidadi et al., 2021 (Alidadi et al., 2021)	66 patients with MetS; randomized, double-blind, placebo-controlled trial	Curcumin 500 mg/day vs placebo	12 weeks	Pulse wave velocity and vascular indices	Improvement in PWV/aortic stiffness after adjustment	No verified ClinicalTrials.gov NCT identified	Adverse events were not reported in the published study; safety assessment therefore remains insufficient.	Surrogate cardiovascular endpoint; no cardiovascular event data; safety reporting limited
Yaikwawong et al., 2024 (Yaikwawong et al., 2024)	272 obese adults with T2D; randomized, double-blind, placebo-controlled trial	Curcumin extract 1500 mg/day vs placebo	12 months	FPG, HbA1c, HOMA-β, HOMA-IR, adiponectin, leptin, BMI	↓ FPG, HbA1c, HOMA-IR, leptin and BMI; ↑ HOMA-β and adiponectin	Thai Clinical Trials Registry: TCTR20140303003	Only mild adverse effects were reported, including abdominal pain, diarrhea, and headache. No participants withdrew because of adverse effects; no serious adverse effects or hypoglycemia were reported, and AST, ALT and creatinine did not differ significantly between groups.	T2D rather than MetS-specific population

NCT, National Clinical Trial identifier registered at ClinicalTrials.gov; TCTR, Thai Clinical Trials Registry. “Not identified” indicates that no ClinicalTrials.gov registration could be reliably linked to the published study after cross-checking the publication details and registry records. Multiple publications arising from the same randomized trial are grouped to avoid double-counting independent clinical evidence.

Safety reporting: “Not reported” indicates that adverse-event information was not sufficiently provided in the original publication and should not be interpreted as evidence that no adverse events occurred.

Overall, the previously mentioned clinical studies corroborate the majority of findings from the cell- and animal-model studies discussed in previous sections, thereby supporting the hypothesis regarding the potential benefits of curcumin in the treatment of MetS.

## 6 Systems biology and multi-omics insights into Curcumin’s effects

A thorough understanding of the pleiotropic effects of curcumin in MetS has been facilitated by emerging systems biology and multi-omics approaches, which have expanded beyond single-target mechanisms to include intricate network interactions. Numerous potential targets of curcumin have been identified through network pharmacology analyses, such as critical nodes in insulin signaling (PI3K/AKT), inflammation (NF-κB, TNF-α, and IL-6), lipid metabolism (SREBP1 and PPARα), and oxidative stress pathways (NRF2/KEAP1). Roney and Mohd Aluwi (2026) conducted a comprehensive mini-review that utilized network pharmacology and systems biology to investigate the multi-target modulation of curcumin in T2D and MetS. The research employed protein-protein interaction networks, Gene Ontology (GO) analyses, and KEGG pathway enrichment to demonstrate that curcumin primarily targets hub genes associated with chronic low-grade inflammation, adipocyte dysfunction, and insulin resistance (Roney and Mohd Aluwi, 2026). Integrated multi-omics studies corroborate these computational findings. Transcriptomic and proteomic analyses in high-fat diet models have shown that curcumin mitigates dysregulation of genes associated with mitochondrial function, fatty acid oxidation, and endoplasmic reticulum stress, and modulates metabolic interactions between the gut microbiota and the host. Network pharmacology, molecular docking, and dynamic simulations have demonstrated the significant binding affinity of curcumin with essential proteins such as AKT1, PPARG, and HMGCR. Pokhrel et al. employed network pharmacology, molecular docking, and molecular dynamics simulations to illustrate the therapeutic potential of curcuminoids in T2D. The analysis demonstrated substantial enrichment in the AMPK, PI3K-AKT, and PPAR signaling pathways, among others, which closely align with preclinical findings and elucidate the extensive metabolic advantages observed in MetS (Pokhrel et al., 2025). Additionally, curcumin’s actions have been further elucidated by recent research that integrates transcriptomics and metabolomics. Curcumin regulates epigenetic factors, including histone acetylation and microRNA expression, that are involved in insulin sensitivity, and it appears to normalize altered metabolite profiles, including branched-chain amino acids, ceramides, and bile acids. These multi-omics approaches also suggest that curcumin affects the gut microbiome-metabolome axis. Specifically, it promotes beneficial bacteria, such as *Akkermansia muciniphila*, while reducing LPS production, thereby mitigating metabolic endotoxemia at the systemic level (Zhong et al., 2022). These integrative analyses provide hypotheses regarding how multiple molecular pathways may contribute to the metabolic actions of curcumin; however, their relevance to the clinical efficacy of specific formulations requires direct validation in human studies (Bateni et al., 2022).

In summary, systems biology and multi-omics analyses position curcumin as a potential multi-target nutraceutical for MetS. Nonetheless, the majority of existing research remains computational or preclinical; subsequent investigations should incorporate human multiomics datasets (e.g., longitudinal transcriptomics, proteomics, and metabolomics) from randomized controlled trials to validate these networks and identify response biomarkers for personalized curcumin therapy. This integrative approach enhances mechanistic understanding of curcumin and guides the development of more effective synergistic and nanoformulated products (Pokhrel et al., 2025; Roney and Mohd Aluwi, 2026).

## 7 Bioavailability, formulations, and translational challenges

An important limitation when interpreting preclinical studies is the frequent use of relatively high curcumin doses, commonly expressed on a mg/kg body-weight basis. Such doses should not be directly compared with human oral doses because species differ substantially in absorption, metabolism, clearance, and body-size scaling. Moreover, administered dose does not necessarily reflect systemic exposure, particularly for curcumin, whose oral bioavailability is strongly influenced by formulation. Therefore, the doses used in animal models should primarily be interpreted in the context of mechanistic proof-of-concept rather than as directly translatable human dosing regimens. The primary challenge in translating the promising preclinical effects of curcumin into reliable clinical benefits for MetS is its inadequate oral bioavailability. Curcumin exhibits low aqueous solubility, undergoes rapid metabolism in the intestine and liver (primarily via glucuronidation and sulfation), and is swiftly excreted from the body, resulting in minimal concentrations of curcumin in the bloodstream and tissues, even at elevated dosages. Classic pharmacokinetic studies indicate that oral administration of standard curcumin at doses of 8-12 g/day yields plasma concentrations that are often below 1 μM, potentially insufficient for optimal therapeutic efficacy across various tissues (Anand et al., 2007; Tabanelli et al., 2021). To overcome these challenges, various advanced formulations have been developed, including piperine-enhanced extracts, phytosomal complexes, liposomes, micelles, and nanoparticles. The co-administration of piperine, derived from black pepper, can increase bioavailability by inhibiting glucuronidation. Significant increases in absorption and tissue distribution, ranging from 10 to 100-fold, have been observed with advanced delivery systems such as nano-micelle and phytosomal curcumin. Co-administration of piperine can enhance bioavailability by inhibiting glucuronidation. Pharmacokinetic studies have reported substantial, formulation-dependent increases in curcumin exposure with several enhanced delivery systems; however, the magnitude of improvement varies considerably among preparations and cannot be generalized across all nano-micellar, phytosomal, or other enhanced formulations (Hegde et al., 2023; Moradi Baniasadi et al., 2025). Bioavailability-enhanced formulations have produced promising findings in several clinical studies; however, differences in populations, doses, formulations, study durations, and endpoints, together with the limited number of direct head-to-head comparisons, currently preclude firm conclusions regarding their clinical superiority over conventional curcumin. Low-dose nano-micelle curcumin (80 mg/day for 8–12 weeks) markedly enhanced glycemic control (fasting blood glucose and HOMA-IR), lipid profile (triglycerides), oxidative stress indicators (decreased MDA, increased TAC), and adiponectin levels, while mitigating inflammation in patients with MetS (Bateni et al., 2022). Wilar et al. demonstrated that nanocurcumin supplementation markedly enhanced anthropometric indices, blood pressure, lipid profiles, and glycemic parameters in patients with MetS (Wilar et al., 2025). Despite these advancements, significant translational challenges remain. Firstly, there exists significant heterogeneity in formulation types, dosages, and study durations, complicating direct comparisons. Secondly, while curcumin is typically acknowledged as safe (GRAS) at elevated doses, infrequent cases of idiosyncratic hepatotoxicity have been documented with enhanced-bioavailability formulations (notably those containing piperine), leading to regulatory advisories from the TGA and Health Canada. Liver enzyme monitoring is advised for prolonged or high-dose usage, particularly in at-risk individuals (Lombardi et al., 2021). Ultimately, the majority of clinical trials are short-duration (≤12 weeks) and have comparatively small sample sizes, with limited long-term cardiovascular outcome data. These constraints necessitate extensive, prolonged head-to-head trials comparing different formulations to conclusively determine curcumin’s role as an adjunct therapy for MetS.

## 8 Conclusion and future direction

MetS is defined by central obesity, hyperglycemia, dyslipidemia, and hypertension, which can markedly elevate the risk of type 2 diabetes and cardiovascular disease. Obesity and insulin resistance are pivotal in the pathogenesis of MetS. Obesity can enhance the infiltration of immune cells into white adipose tissue and elevate the secretion of cytokines such as TNF-α, potentially resulting in low-grade systemic inflammation. TNF-α can impair insulin signaling through multiple mechanisms and induce insulin resistance in metabolically significant tissues, potentially resulting in hyperglycemia. Obesity can diminish adiponectin levels, which have anti-inflammatory properties, while elevating leptin levels, which have pro-inflammatory properties. A high-fat diet and obesity can impair the intestinal mucosal barrier and alter the composition of gut microbiota, resulting in elevated circulating levels of LPS, which subsequently fosters low-grade systemic inflammation and may exacerbate insulin resistance. Chronic mild psychological stress can disrupt the hypothalamus-pituitary axis and elevate glucocorticoid levels, resulting in insulin resistance and hyperglycemia. Inflammation can stimulate lipolysis in white adipose tissue, resulting in increased release of free fatty acids and elevated concentrations in the bloodstream. Tissues, such as the liver, absorb these free fatty acids. Elevated intracellular fatty acid levels can induce oxidative stress, subsequently impairing insulin signaling. Moreover, oxidative stress and inflammation mutually enhance one another. An elevation in hepatic fatty acid levels enhances VLDL production, consequently raising plasma triglyceride levels. Elevated VLDL levels in circulation are associated with increased triglyceride exchange with HDL via CETP, potentially reducing HDL levels. Oxidative stress adversely affects blood vessels, elevating the release of angiotensin II from white adipose tissue and augmenting the expression of its receptors in obesity and insulin resistance, potentially leading to elevated blood pressure. Curcumin’s ability to influence numerous critical mechanisms involved in metabolic processes has been clearly demonstrated in cellular and animal models. These studies suggest that curcumin may attenuate adipogenesis while promoting thermogenesis and energy expenditure, thereby effectively reducing weight gain. Furthermore, curcumin has been demonstrated to improve hyperglycemia, alleviate oxidative stress, elevate adiponectin levels, decrease LPS levels, enhance insulin signaling, reduce insulin resistance, and lower leptin levels. This article evaluates research on animal and cell models, indicating that curcumin has the potential to reduce cortisol levels and possibly alleviate depressive-like behaviors. Results suggest that curcumin not only inhibits lipogenesis in liver tissue and decreases VLDL production but also reduces lipolysis in white adipose tissue and lowers circulating free fatty acid levels. In contrast, curcumin has the potential to significantly increase HDL levels by promoting its synthesis in the liver, facilitating reverse cholesterol transport, and inhibiting CETP activity. Additionally, curcumin may enhance the management of hypertension. Its antioxidant properties, enhancement of eNOS, and reduction of ATR1 expression are likely responsible for its antihypertensive effects. Human clinical studies provide partial support for some of these mechanistic observations, particularly with respect to selected glycemic, lipid, inflammatory, oxidative-stress, and anthropometric outcomes. However, the magnitude and consistency of these effects vary across studies, and most available trials have evaluated surrogate endpoints rather than long-term clinical outcomes. Most of these studies suggest that taking curcumin can help people with MetS lose weight, reduce insulin resistance, improve dyslipidemia, and lower blood sugar levels. Few studies have examined curcumin’s effects on hypertension in MetS, and most clinical trials initially enrolled patients with normal blood pressure. However, certain studies have reported that curcumin supplementation has beneficial effects on systolic blood pressure in overweight individuals with suboptimal plasma glucose levels.

Overall, preclinical studies provide substantial mechanistic evidence that curcumin can modulate several interconnected processes involved in MetS, while human studies provide partial and heterogeneous support for improvements in selected cardiometabolic risk markers. However, most clinical trials are relatively small and short in duration and have primarily assessed surrogate rather than hard clinical endpoints. Poor and formulation-dependent bioavailability further complicates translation of preclinical findings to clinical practice. Although enhanced formulations may increase systemic exposure, their clinical superiority over conventional curcumin has not been established in direct comparative trials. Future studies should therefore prioritize adequately powered, longer-duration randomized trials using well-characterized formulations, standardized outcomes, careful safety monitoring, and clinically meaningful endpoints.

## Authors’ contributions

Mostafa Vaghari-Tabari, Abdolkarim Mahrooz, and Asal Golchin wrote the article; Mehran Molavand prepared the figures and tables; Bahman Yousefi designed and revised the article. All the authors studied and approved the final manuscript. The authors declare that all data were generated in-house and that no paper mill was used.

## Artificial intelligence use statement

The authors declare that ChatGPT (OpenAI, San Francisco, CA, USA) was used solely for language editing and improving the readability of the manuscript. The tool was not involved in the generation of scientific content, data analysis, or interpretation of results.

## Funding

This research did not receive any specific grant from funding agencies in the public, commercial, or not-for-profit sectors.

## Declaration of competing interest

The authors declare that they have no known competing financial interests or personal relationships that could have appeared to influence the work reported in this paper.

## Data Availability

No data was used for the research described in the article.
